# Exploration of the Global Minimum and Conical Intersection with Bayesian Optimization

**DOI:** 10.1002/minf.202400041

**Published:** 2025-01-30

**Authors:** Riho Somaki, Taichi Inagaki, Miho Hatanaka

**Affiliations:** ^1^ Graduate School of Science and Technology Keio University 3-14-1, Hiyoshi Kohoku-ku, Yokohama 223-8522 Japan; ^2^ Institute for Molecular Science 38 NishigoNaka Myodaiji, Okazaki 444-8585 Japan

**Keywords:** derivative-free, gaussian process regression, geometry optimization

## Abstract

Conventional molecular geometry searches on a potential energy surface (PES) utilize energy gradients from quantum chemical calculations. However, replacing energy calculations with noisy quantum computer measurements generates errors in the energies, which makes geometry optimization using the energy gradient difficult. One gradient‐free optimization method that can potentially solve this problem is Bayesian optimization (BO). To use BO in geometry search, an acquisition function (AF), which involves an objective variable, must be defined suitably. In this study, we propose a strategy for geometry searches using BO and examine the appropriate AFs to explore two critical structures: the global minimum (GM) on the singlet ground state (S_0_) and the most stable conical intersection (CI) point between S_0_ and the singlet excited state. We applied our strategy to two molecules and located the GM and the most stable CI geometries with high accuracy for both molecules. We also succeeded in the geometry searches even when artificial random noises were added to the energies to simulate geometry optimization using noisy quantum computer measurements.

## Graphical abstract

We proposed the Bayesian optimization scheme to locate the global minimum and the most stable conical intersection, which could open the door to the geometry optimization using the noisy intermediate‐scale quantum (NISQ) devices.

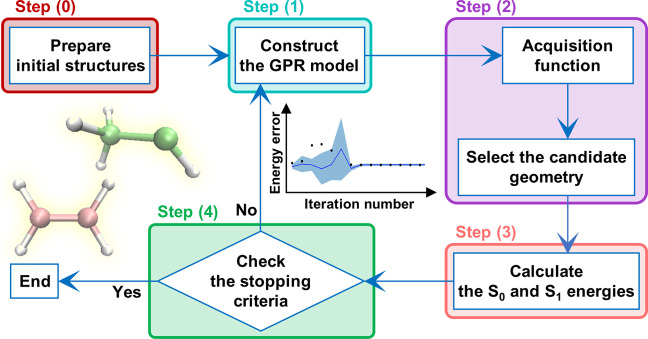

## 1 Introduction

Geometry optimization is an essential technique for studying the chemical properties of molecules. Conventional geometry optimization uses energy gradients obtained from quantum mechanical (QM) calculations [1, 2, 3]. Geometry optimization using energy gradients is applicable not only to the global minimum (GM) but also to local minima (LMs), transition states (TSs), and conical intersections (CIs). In recent years, high‐precision QM calculations using quantum computers have attracted attention as a method for large‐scale QM calculations in the full configurational interaction levels [4, 5, 6, 7, 8, 9]. However, current quantum computers, called noisy intermediate‐scale quantum (NISQ) computers, are not sufficiently advanced for fault tolerance, and their measured energies contain errors [10, 11]. Therefore, the potential energy surfaces (PESs) described with such energies have non‐smooth shapes, which makes geometry optimization using energy gradients very difficult. In addition, for some highly accurate QM calculations (without quantum computers), an analytical gradient technique is not implemented, which makes geometry optimization difficult owing to the high computational cost of obtaining numerical gradients. Therefore, a new geometry search method without energy gradients is urgently needed. One gradient‐free optimization method that can potentially solve this problem is Bayesian optimization (BO) [12].

BO is a black‐box optimization method that searches for a solution with the desired properties by repeatedly constructing a regression model and proposing the data points to be obtained. In BO, the mean *μ*(***x***) and variance *σ* ^2^(***x***) of the predicted objective variable *f*(***x***) for each data point ***x*** are calculated using Gaussian process regression (GPR) [13]. The mean tells us whether the prediction result at each data point is close to the desired property. From the variance, we can obtain information on the prediction uncertainty at each data point. In BO, this information is used in a balanced manner to select the candidate data points. “Exploitation” and “exploration” are important concepts in the selection of candidate data points. Exploitation is the concept of selecting candidate points whose predicted values are close to the target (desired value) from the currently available data. Exploration, on the other hand, is the concept of selecting candidate points where the variance is high, *i. e*., where limited data have been sampled. Their balance is determined by the acquisition function (AF) used in BO. One conventional AF is the probability of improvement (PI) [14], which represents the probability that the predicted value *f*(***x***) at data point ***x*** is greater than the maximum already found *f*(***x*** *), as follows:

(1) $$\mathrm{PI}\left(x\right)=P\left(f\left(x\right)\geq f\left(x^{*}\right)\right)=\Phi \left(\frac{f\left(x^{*}\right)-\mu \left(x\right)}{\sigma \left(x\right)}\right)$$

where *P*, *Φ*, *μ*(***x***), and *σ*(***x***) are the probability, cumulative distribution function, mean, and standard deviation, respectively, calculated using the GPR model. Another widely used AF is the upper confidence bound (UCB) [15], which is the weighted sum of the mean *μ*(***x***) and standard deviation *σ*(***x***), as follows:

(2) $$\mathrm{UCB}\left(x;\;\beta \right)=\mu \left(x\right)+\sqrt{\beta }\sigma \left(x\right)$$

Here *β* is a hyperparameter that determines the balance of exploitation and exploration.

To the best of our knowledge, only a few applications of BO in molecular geometry searches have been reported. Fang *et al*. explored amino acid conformers using BO, followed by conventional geometry optimization at the QM level [16]. Similarly, Chan *et al*. applied BO to find the most stable conformer by optimizing the dihedral angles and found that BO required far fewer energy calculations than systematic or random searches [17]. They reformulated the lowest conformer search as a constrained BO problem and successfully found lower conformers than those in their previous study [17] using knowledge‐based expected improvement as an AF [18]. Recently, Bae *et al*. optimized all degrees of freedom in supported metal systems by BO [19]. They proposed a hybrid approach that combining BO and conventional geometry optimization and succeeded in finding the most stable structure efficiently. In these studies, the target structures were only the LMs on the ground state, and a BO strategy for exploring other critical structures, such as TSs and CIs, is not proposed.

In this study, we propose a strategy to search for the GM geometry on the ground state and the most stable CI geometry between the ground and excited states using BO. In Section 2, we outline the calculation scheme of the geometry search method for the GM and CI structures using BO. A method validating the accuracy of the proposed BO scheme is also described. In Section 3, we present the results of applying our BO scheme to the search for the GM and CI structures of the two molecules, formaldehyde and ethylene. The appropriate AFs and objective variables for GM and CI searches are discussed. Finally, we present the results when artificial random noise is added to the energies to simulate geometry optimization using noisy quantum computer measurements.

## 2 Computational Details

### 2.1 Calculation Scheme for the Geometry Exploration

**Figure 1:**
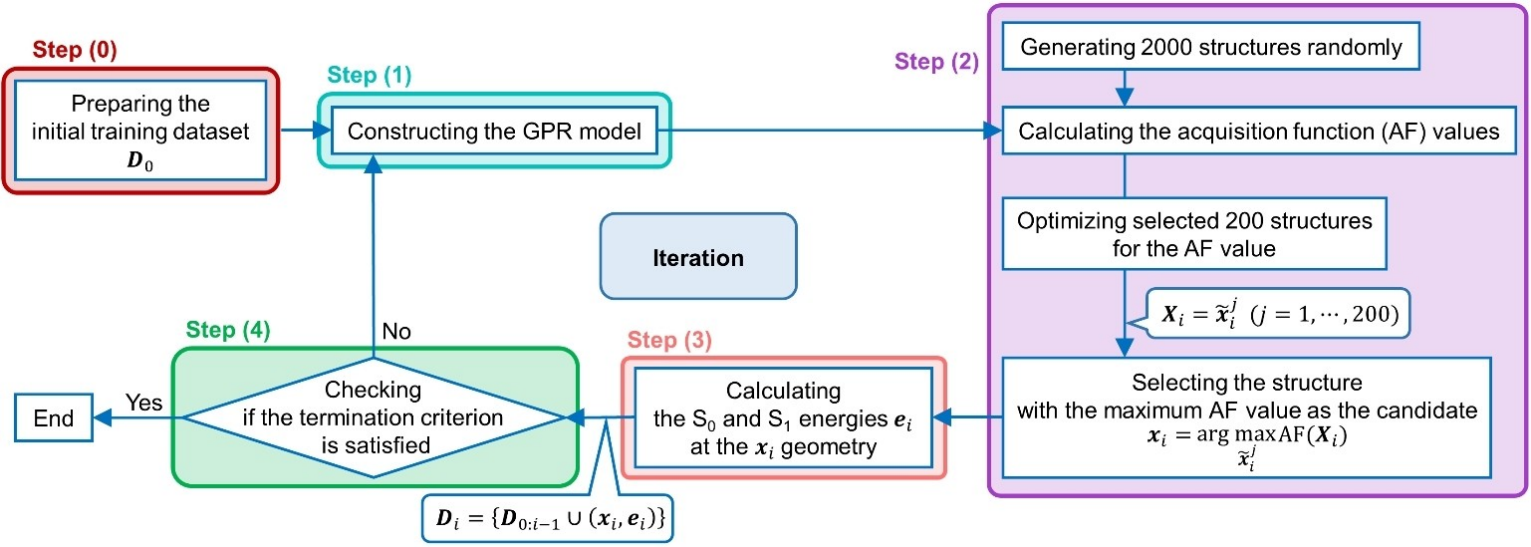
Overview of our molecular structure search method with BO, featuring the (0) preparation of the initial training dataset ***D***_0_, (1) construction of the GPR model, (2) identification of the geometry with the maximum AF value as the candidate geometry, (3) energy calculation at the DFT or TDDFT level, and (4) checking if the termination criterion is satisfied. ***D***_0:*i*‐1_, ***X**_i_*, ***x**_i_*, ***e**_i_* represent the training dataset used for constructing the GPR model at the *i‐*th iteration, optimized 200 geometries for the AF value, the candidate geometry, and its energy, respectively.

As shown in Figure 1, our geometry search method with BO involves five steps: preparation of the initial training dataset (step 0), construction of a GPR model (step 1), identification of the geometry with the maximum AF value as the candidate geometry (step 2), energy calculation at the QM level (step 3), and checking the termination criterion (step 4). Steps 1–4 are repeated until the termination criterion is satisfied. The details of each step are as follows:

In step 0, the initial training dataset for a GPR model is prepared. This dataset consists of molecular structures and their energies. To collect a wide variety of structures, any method with a low computational cost is applicable. In this study, we used an automated reaction path search method called the single‐component artificial force‐induced reaction (SC‐AFIR*n*) method [20, 21], in which up to *n* force functions were applied simultaneously, with the energy of the singlet ground state (S_0_) and its gradient obtained using the GFN2‐xTB method [22]. In the case of formaldehyde, the SC‐AFIR2 method [20, 21, 23] found 21 reaction pathways. From each of the 21 reaction pathways, distinct structures were extracted based on their S_0_ energies as shown in Figure S1. We excluded structures with atom pairs closer than 0.5 Å, because the energies of these structures could be extremely high. This procedure gave us a total of 71 unique structures. For ethylene, the SC‐AFIR calculations collected 14 reaction pathways. Following the same procedure as that for formaldehyde, we obtained 41 unique structures. The initial structures used for the SC‐AFIR and SC‐AFIR2 calculations were GMs optimized at the GFN2‐xTB level of theory. The parameters for the SC‐AFIR and SC‐AFIR2 were set as follows: the collision energy parameter *γ* was 500 kJ mol^−1^, the parameters for the termination criterion, such as SCGO_mLatest and SCGO_nLowest were 20, and the NoBondRearrange option was used. SC‐AFIR and SC‐AFIR2 calculations were performed using the GRRM program [24, 25], with GFN2‐xTB energies and their gradients using the Orca program [26]. We used the SC‐AFIR method because it was the most convenient for us to collect a wide range of geometries in a short time. Other methods, such as molecular dynamics simulations and random generation, may be applied. The energy at each geometry obtained in the above procedure was computed. The electronic energies of S_0_ and the first singlet excited state S_1_, denoted *E*(S_0_) and *E*(S_1_), respectively, were calculated using the density functional theory (DFT) and time‐dependent (TD) DFT levels. The DFT and TDDFT calculations were performed with the ωB97XD functional [27] and the cc‐pVDZ basis set [28] using the Gaussian 09 program [29].

**Figure 2:**
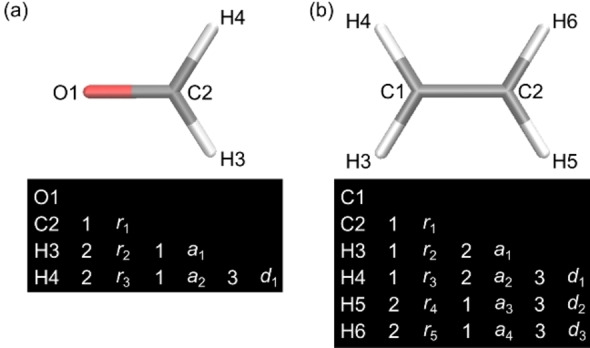
The definition of the Z‐matrixes for formaldehyde (a) and ethylene (b) with the atom indexes and internal coordinates used in the Z‐matrixes. Carbon, oxygen, and hydrogen atoms are shown in gray, red, and white, respectively.

In step 1, a GPR model for predicting electronic energy is constructed. The explanatory variables were the internal coordinates defined in Figure 2. There were six explanatory variables for formaldehyde (three distances, two angles, and one dihedral angle) and 12 for ethylene (five distances, four angles, and three dihedral angles). The objective variables depend on the target structure. The objective variable for the GM search was −*E*(S_0_) to replace the minimization problem with the maximization problem. The objective variable for the most stable CI search, on the other hand, was uncertain because the CI geometry is the minimum energy point that satisfies the energy gap condition *E*(S_1_)−*E*(S_0_)=0. To consider the two conditions; (1) the S_0_ and S_1_ energies were degenerate, and (2) their energies were minimized, we propose the use of a cost function *C*, which is the weighted sum of the average and squared gap of *E*(S_0_) and *E*(S_1_), as follows:

(3) $$C=\frac{E\left(S_{0}\right)+E\left(S_{1}\right)}{2}+\frac{{\left(E\left(S_{1}\right)-E\left(S_{0}\right)\right)}^{2}}{\alpha }$$

Here, *α* is a hyperparameter that determines the weight of the constraint on the energy degeneracy of S_0_ and S_1_. This cost function with a fixed *α* was originally used for the geometry estimation of the CI, followed by geometry optimization, where *α* gradually decreased until the energy gap between S_0_ and S_1_ became smaller than the threshold [30, 31]. As in the GM search, we need to replace the minimization problem with the maximization problem, thus −*C* was applied to the objective variable for the search of the most stable CI. Note that in our preliminary study for the CI search, other objective variables and AFs were also examined, as shown in Table S1. However, none of their BO campaigns satisfied the termination criterion. The details of the GPR model construction were as follows: The radial basis function (RBF) kernel [13] was applied, the Adam optimizer [21] was used with a learning rate of 0.2, and 100 epochs were selected. All the explanatory and objective variables were standardized. The model was constructed using the GpyTorch [33] and BoTorch packages [34].

In step 2, to propose the candidate geometry, we generated many geometries and extracted the geometry with the maximum AF value. The AFs examined were the PI [14] and the UCB [15]. Due to the vast search space, calculating the AF values for all possible geometries is difficult. Therefore, we applied the following procedure: First, 2000 random structures were generated within a defined search space. For formaldehyde, the search space was defined as follows: 0.5 Å≤*r*_1_≤2.0 Å, 0.5 Å≤*r*_2_, *r*_3_≤2.5 Å, 0°≤*a*_1_, *a*_2_≤180°, −180°≤*d*_1_≤180°. For ethylene, the space was 0.5 Å≤*r*_1_≤2.0 Å, 0.5 Å≤*r*_2_, *r*_3_, *r*_4_, *r*_5_≤2.5 Å, 0°≤*a*_1_, *a*_2_, *a*_3_, *a*_4_≤180°, −180°≤*d*_1_, *d*_2_, *d*_3_≤180°. Figure  shows the definition of the internal coordinates. These search spaces can sufficiently describe the C−H bond rearrangements and C−C or C−O bond dissociation. Subsequently, we calculated the AF values of random 2000 structures and selected 200 structures, as implemented in BoTorch (see Figure S2 for the detailed algorithm and Figure S3 for the effect of the number of randomly generated structures). These 200 structures were locally optimized for the AF value with L‐BFGS‐B algorithm [35, 36]. Among the optimized geometries, the one with the maximum AF value was selected as the candidate geometry. If the candidate geometry involved atom pairs whose distances were smaller than 0.5 Å, we excluded this geometry from consideration because excessively unstable structures could negatively affect the GPR model accuracy. Thus, in such cases, we selected the geometry with the maximum AF value among the geometries without atomic collisions. If all the 200 geometries had atomic collisions, the same geometry generation scheme in step 2 was repreated.

In step 3, the energy at the candidate geometry selected in step 2 is computed. *E*(S_0_) and *E*(S_1_) were calculated at the same level as in step 0.

In step 4, we check whether the optimization termination criterion is satisfied. The criterion for the GM search was as follows: Whenever the iteration number of steps 1 to 3 was a multiple of five, the most stable geometry among all candidates was stored. If the most stable geometry was not updated in three consecutive checks, the optimization was terminated. In other words, the optimization was terminated when no more stable geometry was not found in three consecutive checks (see Figure S4 for the schematic illustration of this termination criterion). The criterion for determining the most stable CI was similar. When the iteration number reached a multiple of 50, we stored the structure with the lowest *E*(S_1_) among the candidates whose energy gap *E*(S_1_)−*E*(S_0_) was less than 3.0 kcal mol^−1^. If the stored structures were not updated in three consecutive checks, the optimization was terminated. Even if the termination criterion was not met, we terminated the optimization when the total number of iterations exceeded 1000.

### 2.2 Evaluation

**Figure 3:**
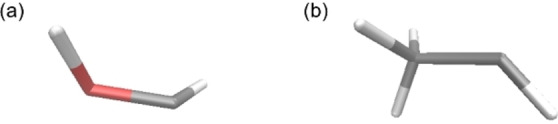
The reference (true) CI geometries of formaldehyde (a) and ethylene (b). The atoms are shown in the same color code as in Figure 2.

To evaluate the performance of our BO procedure, we conducted three optimization campaigns for each optimization target/condition because step 2 involved randomness. The difference between the obtained and reference (true) geometries was estimated using the electronic energy difference and the root mean square deviation (RMSD) of the atomic positions. The reference GM geometries were obtained *via* conventional geometry optimization at the ωB97XD/cc‐pVDZ level. To obtain the reference geometries of CI between S_0_ and S_1_, it is well known that the TDDFT is not appropriate due to the discontinuous of the PESs close to the CI. To solve this problem, several methods were proposed including the spin‐flip TDDFT [37, 38] and the energy shift approximation [39]. In this study, we applied the DFT and TDDFT calculations at the ωB97XD/cc‐pVDZ level combined with the energy shift approximation. In this approach, the PES of S_1_ is shifted downward to avoid discontinuities on the PES calculated by TDDFT near the CI structure. This also shifts the CI point downward, allowing the CI structure to be optimized without entering the discontinuous region. The energy shift parameter for S_1_ was set to 0.2 kcal mol^−1^. The CI geometries were exhaustively explored using the SC‐AFIR method. The most stable CI geometries of formaldehyde and ethylene were hydrogen‐transfer‐type and ethylidene‐like geometries, respectively (Figure 3). All geometry optimizations of the reference structures were performed using the GRRM program, with the energies and their gradients obtained from the Gaussian09 program. The most stable CI geometry of formaldehyde has two conformational isomers with a mirror‐image relationship. Thus, the smaller RMSD for the two true conformational isomers was used to evaluate the structures obtained using BO.

## 3 Results and Discussion

### 3.1 Application to Formaldehyde

**Figure 4:**
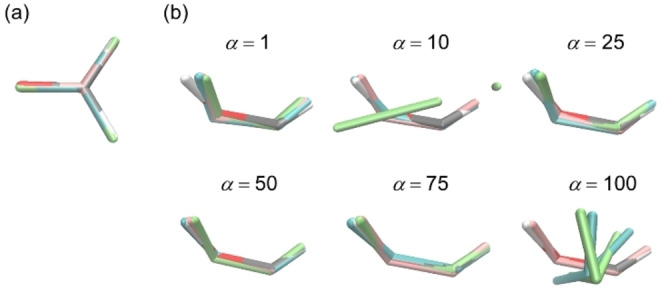
Comparison between the reference geometry (in the same atom color code as in Figure 2) and the geometries obtained *via* the first, second, and third BO campaigns (in pink, blue, and green, respectively) in the GM search (a) and the most stable CI search using different *α* (in kcal mol^−1^) parameters (b) of formaldehyde.

First, we performed a BO geometry search for the GM of formaldehyde. Table 1 lists the results of the three optimization campaigns, all of which successfully obtained geometries with very small energy differences and RMSDs from the reference GM. A comparison of the three obtained geometries with the reference GM, as shown in Figure 4a, also confirmed that the three optimization campaigns yielded geometries very close to the reference GM.

**Table 1:** Results of the GM search of formaldehyde.

Entry^[a]^	Iteration number^[b]^	Energy difference Δ*E*(S_0_) (kcal mol^−1^)^[c]^	RMSD (Å)^[c]^
1	30 (19)	0.207	0.0111
2	30 (19)	0.555	0.0173
3	20 (10)	0.643	0.0242

^[a]^ We performed three optimization campaigns because the BO procedure involves randomness in step 2. ^[b]^ Number of iterations until termination of BO. The numbers in parentheses are the iteration numbers that yielded the lowest‐energy geometry. ^[c]^ The energy difference Δ*E*(S_0_) and RMSD were calculated from the reference GM structure.

**Figure 5:**
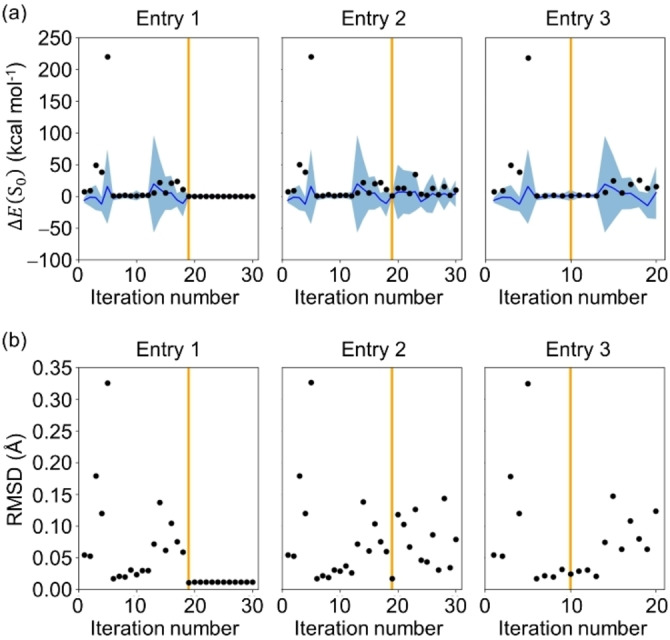
The deviation of the candidate geometry from the reference GM geometry of formaldehyde was estimated by the energy difference Δ*E*(S_0_) (in kcal mol^−1^) (a) and the RMSD (in Å) (b) at each iteration for entries 1, 2 and 3 in Table 1. The Δ*E*(S_0_) values calculated with the DFT and predicted from the GPR model are represented by black dots and a blue line, respectively. The 68% confidence interval of the GPR model is in light blue. The orange lines represent the iteration number that yielded the optimized geometry. The absolute Δ*E*(S_0_) in log scale and the evolution of the mean absolute error (MAE) on the *E*(S_0_) prediction of all future candidates are also shown in Figure S5.

**Figure 6:**
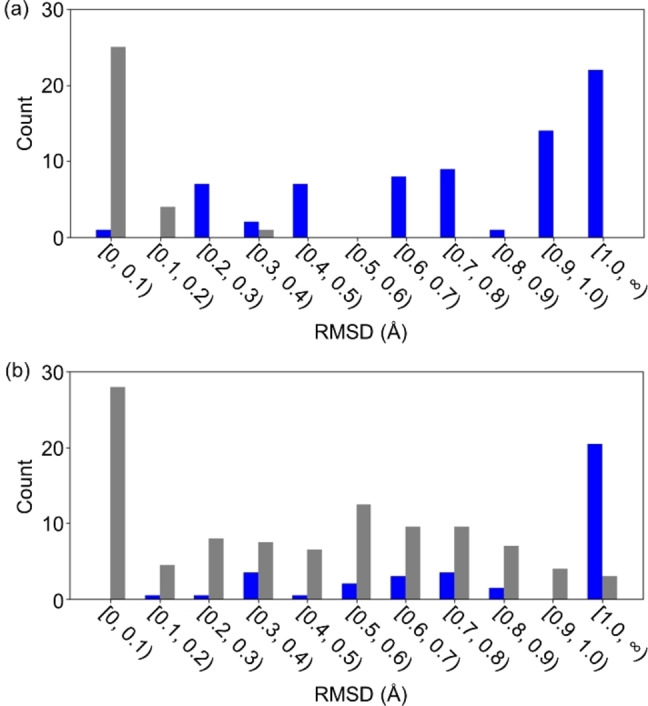
Histograms of the RMSD (in Å) for the geometries used through the GM search (entry 1 in Table 1) (a) and the CI search (entry 10 in Table 2) (b). The RMSDs are calculated from the reference GM and CI geometries. The initial and candidate geometries are in blue and gray, respectively. The initial geometries used for (a) and (b) are identical.

To understand how BO worked, the deviation between the reference and candidate geometry generated at each iteration was analyzed using the energy difference and RMSD. As shown in Figure 5, geometries with extremely large variances were selected as candidates in the early stages of BO, which indicated that exploration was preferred to exploitation. When exploration was favored, the geometries far from the initial geometry set, such as those involving atom pairs with short distances, had a higher chance of selection. However, their energies were extremely unstable, resulting in a decrease in their AF values after a few iterations. Thus, the significantly unstable geometries were no longer selected only after about 5 iterations. Indeed, relatively stable geometries were more likely to be selected after 5 iterations as shown in Figure 5. Unlike conventional geometry optimization using energy gradients, a search for regions far from the true structure can be performed even after the regions near the true structure have been explored (see, for example, iteration numbers 13–18 in Entry 1 in Figure 5). The reason for the switch from the exploitation to exploration can be understood by the overfitting to the data close to the true structure. As the exploitation continued, the energy prediction accuracy increased for the geometries close to the true GM, but decreased for those far from the true GM, which implied that the GPR model became overfitted to the data close to the true GM (see Figure S5). This tendency to overfit was observed in all the three BO campaigns and could be a serious problem when describing a PES. However, since our goal is to search for a critical structure, we are not interested in regions far from the true geometry. Therefore, overfitting in the vicinity of the true GM serves our purpose. Furthermore, one of the reasons for the stable success of the GM optimization for all three optimization campaigns could be attributed to the fact that the initial structure set included data close to the reference GM (RMSD=0.013 Å). As shown in Figure 6a, the initial training data involved only one geometry whose RMSD from the reference was less than 0.20 Å, and then the data close to the reference GM were collected frequently during the BO.

Additionally, we examined another AF, the UCB in Equation (2), for the GM search. In contrast to the PI, the UCB involved the hyperparameter *β*, which significantly affected the results. Among the *β* values examined in Table S2, we were able to locate the geometry close to the reference GM geometry for the three BO campaigns only when *β* was set to 0.1.

Second, we focused on the most stable CI search using the cost function in Equation (3). To investigate the effect of the parameter *α*, BO with *α=* 1, 10, 25, 50, 75, and 100 kcal mol^−1^ was performed three times each, as shown in Table 2 (see Figure 4b for geometries). The averaged Δ*E*(S_0_) over three campaigns were 23.7, 10.3, 4.1, 0.9, 1.0, and 28.1 kcal mol^−1^, for *α*=1, 10, 25, 50, 75, 100 kcal mol^−1^, respectively, indicating that optimization performance heavily depended on *α* and the best was obtained with *α*=50 kcal mol^−1^. By contrast, the energy gap *E*(S_1_)‐*E*(S_0_) were insensitive to *α*. The gaps were 1.9, 2.0, 1.4, 1.9, 1.3, 2.5 kcal mol^−1^, for *α*=1, 10, 25, 50, 75, 100 kcal mol^−1^, respectively.

**Table 2:** Results of the most stable CI search of formaldehyde.

Entry	α (kcal mol^−1^)	Optimization campaign^[a]^	Iteration number^[b]^	Energy difference Δ*E*(S_0_) (kcal mol^−1^)^[c]^	Energy difference Δ*E*(S_1_) (kcal mol^−1^)^[d]^	Energy gap *E*(S_1_)−*E*(S_0_) (kcal mol^−1^)	RMSD (Å)^[e]^
1	1	1	250 (135)	21.048	21.761	0.904	0.1875
2		2	250 (114)	22.204	24.878	2.865	0.1732
3		3	250 (145)	27.863	29.590	1.918	0.2214
4	10	1	300 (175)	3.337	5.949	2.803	0.1151
5		2	350 (231)	10.335	12.433	2.289	0.1061
6		3	300 (173)	17.371	18.018	0.838	1.2026
7	25	1	400 (279)	0.449	2.315	2.057	0.0510
8		2	200 (81)	4.588	5.497	1.101	0.1125
9		3	150 (45)	7.129	8.035	1.097	0.1336
10	50	1	200 (85)	0.448	2.781	2.524	0.0297
11		2	550 (437)	0.648	2.169	1.712	0.0233
12		3	300 (160)	1.574	2.702	1.319	0.0908
13	75	1	250 (127)	0.509	2.728	2.410	0.0524
14		2	250 (132)	1.161	1.750	0.780	0.0423
15		3	250 (106)	1.357	1.777	0.611	0.0418
16	100	1	200 (76)	2.096	3.850	1.944	0.0499
17		2	750 (633)	30.347	32.790	2.633	0.8254
18		3	650 (516)	51.797	54.503	2.897	0.6815

^[a]^ We performed three optimization campaigns because the BO procedure involves randomness in step 2. The optimization campaign IDs (1, 2, and 3) were named in order of decreasing the Δ*E*(S_0_). ^[b]^ Number of iterations until termination of BO. The iteration numbers that yielded the lowest *C* geometry are in parentheses. ^[c]^ Δ*E*(S_0_) was calculated from the *E*(S_0_) at the reference CI structure. ^[d]^ Δ*E*(S_1_) was calculated from the *E*(S_1_) at the reference CI structure. ^[e]^ The smaller RMSD was selected considering the two mirror‐symmetric reference structures.

The detail results with different *α* were focused on. For *α*=1 and 10 kcal mol^−1^, the obtained structures had small energy gaps, but their *E*(S_0_) and *E*(S_1_) were more than 10 kcal mol^−1^ less stable than those of the reference CI geometry in most cases. This could be attributed to the excessive weight of the constraint for energy degeneracy. Using larger *α* values, such as 25, 50, and 75 kcal mol^−1^, geometries quite close to the reference CI geometry were obtained in most cases. The most accurate CI geometry was obtained *via* the BO with *α*=50 kcal mol^−1^ (see entry 10 in Table 2). In spite of appropriate *α* in the range of 25 to 75, BO campaigns, such as entries 8 and 9 in Table 2, resulted in the geometries with relatively large deviations (see Figure 4b), which could be attributed to our loose termination criterion. Even for other optimization campaigns that yielded relatively good results, the differences from the reference geometry in most cases were larger than those from the GM search. This could be attributed to two factors. First, the S_1_ PES calculated at the TDDFT level near the CI geometry is discontinuous. Considering the fact that the reference CI geometry was obtained by the approximation, called the energy shift method [39], to avoid the discontinuous region, it is a good enough result that one of the three optimization campaigns achieved an energy difference of less than 0.6 kcal mol^−1^ (for *E*(S_0_)) from the reference. Second, the CI geometry and its surroundings were not included in the initial structure set. As shown in Figure 6, the initial structure set included the data whose RMSD from the GM reference was only 0.013 Å, whereas the reference CI was relatively far from any initial structure (the smallest RMSD was 0.121 Å.) Thus, it can be inferred that the number of iterations increased compared to the GM search because the search must be performed more widely. A structure closer to the reference CI can be obtained by using a tighter optimization termination criterion. Nevertheless, we emphasize that the current optimization termination criterion is sufficient because the obtained structures are enough for a qualitative discussion. As the *α* value further increased to 100 kcal mol^−1^, the chances of obtaining unstable structures increased, although accurate structure was sometimes obtained, as in the case of entry 16. The effect of *α* can be also understood from the *E*(S_0_) and *E*(S_1_) of the candidate geometries. As shown in Figure S6, with *α*=1 kcal mol^−1^, the candidate geometries were nearly degenerated in energy, but their energies were quite high, which resulted in the very rarely search for geometries with low energies. As *α* increased, the energy gap gradually increased, instead of allowing lower energy geometries to be selected. As the value of *α* was further increased, such as *α*=100 kcal mol^−1^, geometries with small energy gaps were rarely selected. Although it was possible to obtain a geometry close to the reference even with a large *α* value, it was better to keep the *α* value between 25 and 75 kcal mol^−1^.

**Figure 7:**
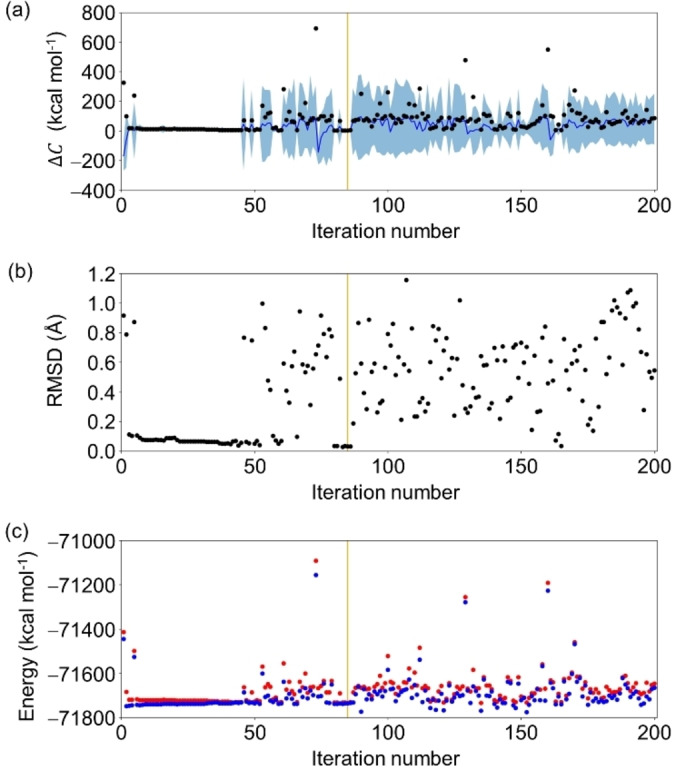
The deviation of the cost function from the reference CI geometry Δ*C* (in kcal mol^−1^) for formaldehyde. The candidate geometries for the CI search were from entry 10 in Table 2. The Δ*C* values calculated by the DFT/TDDFT level and the GPR model are in black and blue, respectively. The 68% confidence interval of the GPR model is in light blue (a). The deviation of the candidate geometry from the reference CI geometry was estimated by the RMSD (in Å) (b) and the S_0_ and S_1_ energies (in blue and red, respectively) at the DFT/TDDFT level (c). The orange lines represent the iteration number that yielded the optimized geometry. The absolute Δ*C* in log scale and the evolution of the MAE on the *C* prediction of all future candidates are also shown in Figure S7.

The optimization process of the best campaign (Entry 10 in Table 2) is shown in Figure 7. At the first stage of the BO, geometries with large variances were selected, then geometries relatively close to the reference structure (RMSD <0.2 Å) tended to be selected. After 50 iterations, the exploration was switched back to exploitation. Thus, in the CI search, candidate geometries were selected from a larger space than in the GM search as shown in Figure 6. The reason for the switch between exploitation and exploration along with the iteration number of BO can be understood from the change in prediction accuracy for the geometries close the reference geometry. As shown in Figure S7, the early stage of the BO (iteration number ≤ 5), the geometries far from the true CI was selected as candidates, which made the prediction accuracy for the geometries close to the true CI worse. After that (iteration number >5), the exploitation tended to be favored to decrease the prediction error of the geometries close to the true CI. And then (iteration number >50), the prediction error for the geometries close to the true geometry became smaller than those far from the true geometry, which induced the switch from exploitation to exploration. Even when the exploration became favored, the prediction error of the geometries close to the true CI kept decreasing.

**Figure 8:**
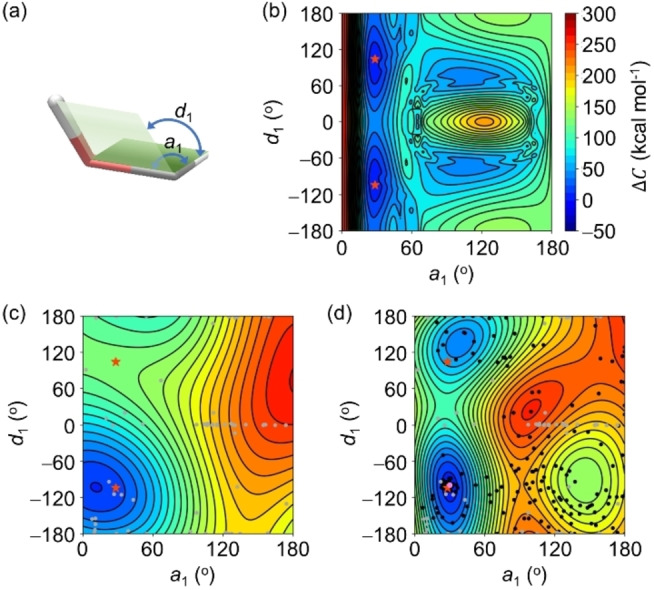
The two internal coordinates (a) used for describing cost function surface (CFS). The CFS measured from the *C* value at the reference CI geometry plotted using the DFT/TDDFT energies (b), predicted values *via* the GPR model constructed using only the initial geometries (c), and predicted values *via* the initial and all the candidate geometries of entry 10 in Table 2 (d). The initial, candidate, optimized geometries are in gray, black, and light pink dots, respectively. The reference geometries are in orange stars.

We investigated how the PES improved as the number of BO iterations increased. Figure 8 shows the cost function surface (CFS) calculated *via* the GPR model and that *via* the DFT/TDDFT calculations as a reference surface. Figure S8 shows the evolution of the CFS as the iteration number of the BO increases. On the reference CFS, calculated using DFT/TDDFT, there were two mirror‐symmetric structures of the most stable CI. Focusing on the CFS calculated by the GPR model constructed using only the initial structure set, the cost function values were relatively high around the CI region (*d*_1_=−104°), owing to the lack of data. As the number of iterations increased, the cost function values of the CI region decreased. Because the BO did not search for both mirror‐symmetric areas equally, only one of the two was obtained as the most stable CI. Our BO procedure cannot search for all multiple solutions (*i. e*., the most stable structures); however, it performed well enough to obtain the most stable geometry.

Next, we applied one of the other AFs, the UCB with the objective variable of −*C*, to determine whether the CI search performance depends on the AF. Unlike the PI, the UCB involves a hyperparameter *β* that controls the balance between exploration and exploitation. Therefore, as shown in Table S3, the two hyperparameters in Equations (3) and (2) (*α* and *β*, respectively) must be tuned, which makes the CI search more difficult. Indeed, only the two parameter sets of *α*=25, 75 kcal mol^−1^ and *β*=0.1 gave accurate CI geometries among all the optimization campaigns. Though other parameter sets sometimes yielded the geometry close to the reference, the optimization performance was not stable.

### 3.2 Application to Ethylene

**Figure 9:**
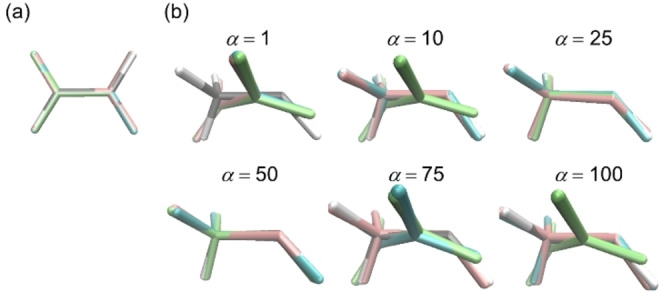
Comparison between the reference geometry (in the same atom color code as in Figure 2) and the geometries obtained *via* the first, second, and third BO campaigns (in pink, blue, and green, respectively) in the GM search (a) and the most stable CI search using different *α* (in kcal mol^−1^) parameters (b) of ethylene.

A similar validation was performed for the geometry optimization of ethylene. Although the degree of freedom of ethylene is 12, twice that of formaldehyde, all three BO campaigns for the GM search successfully located stable structures with a relatively small number of iterations (see Table S4). The deviations of the three obtained geometries from the reference GM were slightly larger than those for formaldehyde, however they were sufficiently close to the reference GM geometry, as shown in Figure 9a. In the case of the CI search, a similar dependence of search performance on the chosen parameter *α* was observed as for formaldehyde. All three BO campaigns failed when the *α* parameter was too small (*α*=1 kcal mol^−1^), as shown in Figure 9b (Table S5). The search performance of the three BO campaigns was stable for *α*=25 and 50 kcal mol^−1^, but RMSD was slightly large in some cases. Also, the results for *α*=1, 10, 75 and 100 kcal mol^−1^ often yielded hydrogen‐migration (HM) structures with RMSDs of approximately 0.65 Å. The HM‐type structure was also a CI geometry with an energy about 5.6 kcal mol^−1^ higher than that of the reference CI (Figure S9). BO sometimes yielded less stable CI structures in the presence of multiple stable CI structures. Although RMSD was slightly large in some cases, we obtained a geometry close to the reference CI by BO with an appropriate *α* parameter (from 25 to 50 kcal mol^−1^).

### 3.3 Effect of Noise in Energy

Finally, we verified that our BO strategy worked well even when the energy calculation involved noise due to the measurements on the NISQ devices. To simulate the noisy measurements, we added artificial random noise to the conventional DFT and TDDFT energies. The random noise was generated from the uniform distribution ranging from −2.0 to 2.0 kcal mol^−1^. The range of the noise was determined based on the calculations on the real quantum device, called the IBMQ‐Kawasaki, by Gocho *et al*. [40].

**Figure 10:**
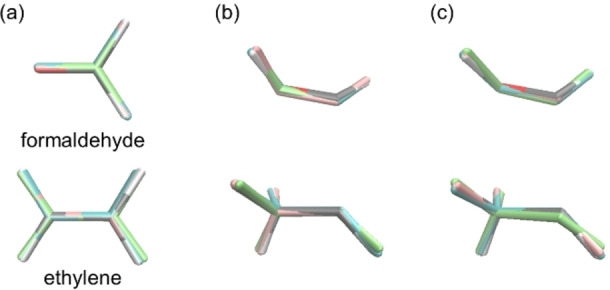
Comparison between the reference geometry (in the same atom color code as in Figure 2) and the geometries obtained *via* the first, second, and third BO campaigns (in pink, blue, and green, respectively) in the GM search (a) and the most stable CI search with *α*=25 kcal mol^−1^ (b), with *α*=50 kcal mol^−1^ (c) of formaldehyde and ethylene. Those with *α*=1, 10, 75, and 100 kcal mol^−1^ are in shown Figure S10.

**Figure 11:**
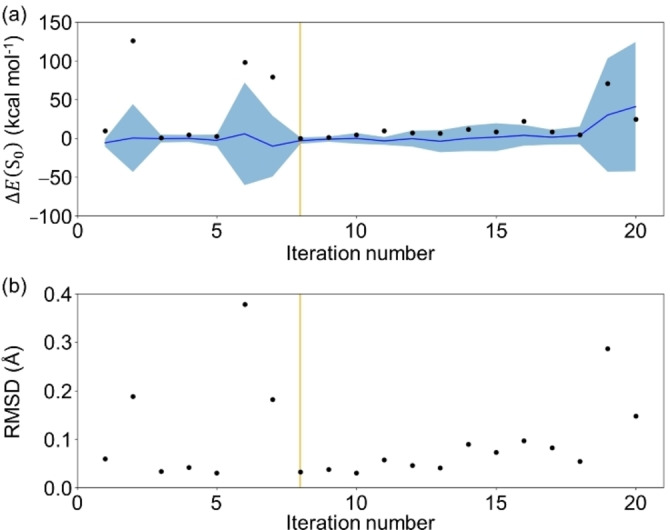
The deviation of the candidate geometry from the reference GM geometry of formaldehyde was estimated by the energy difference Δ*E*(S_0_) (in kcal mol^−1^) (a) and the RMSD (in Å) (b) at each iteration for entry 1 in Table S6. The Δ*E*(S_0_) values calculated with the DFT and predicted from the GPR model are represented by black dots and a blue line, respectively. The 68%confidence interval of the GPR model is in light blue. The orange lines represent the iteration number that yielded the optimized geometry. The absolute Δ*E*(S_0_) in log scale and the evolution of the MAE on the *E*(S_0_) prediction of all future candidates are also shown in Figure S11.

The GM searches with artificial random noise were performed three times each for formaldehyde and ethylene. For both molecules, all three BO campaigns succeeded in locating structures sufficiently close to the reference GM (see Figure 10a) within the energy difference of 1.5 kcal mol^−1^ with relatively small numbers of iterations as shown in Tables S6 and S7. The optimization process of the best campaign for formaldehyde is shown in Figure 11. Though the standard deviations of the candidate geometries with noise were slightly larger than those without noise (see Figure 5), the overall characteristics of the optimization processes were quite similar with and without noise.

The geometry optimization of the most stable CI was also carried out for formaldehyde and ethylene with the hyperparameter *α* was set to 1, 10, 25, 50, 75, and 100 kcal mol^−1^. As shown in Figure 10, the BO successfully located the geometries close to the reference CI geometry for all the campaigns with setting *α*=25 and 50 kcal mol^−1^. The energy differences in *E*(S_0_) were also quite small with the range from 0.5 to 5.5 kcal mol^−1^ for formaldehyde and from 0.2 to 2.6 kcal mol^−1^ for ethylene (see Tables S8 and S9). The optimizations with too small or too large *α* tended to fail to locate the most stable CI, which indicated that the effect of *α* in BO was almost the same regardless of the presence or absence of noise. The performance of CI optimization campaign for formaldehyde shown in Figure S12 was also similar to that in the absence of the noise in Figure 7. From the above, we can say that our BO scheme can locate the GM and the most stable CI with sufficient accuracy even in the presence of noise in energy.

## 4 Conclusions

We proposed a geometry search strategy using BO, a gradient‐free optimization, to find the GM and the most stable CI geometries and applied the strategy to formaldehyde and ethylene. A suitable AF for the GM search was the PI where the objective variable was the electronic energy of the ground state with the opposite sign, −*E*(S_0_). All the optimization campaigns for both molecules achieved quite accurate geometries whose energy deviations and RMSDs from the reference GMs were less than 1.7 kcal mol^−1^ and 0.04 Å, respectively. To search for the most stable CI geometry, we defined the AF as the PI where the objective variable was the weighted sum of the average and squared gap of the ground and excited state energies. The optimization performance depended on the hyperparameter *α*, which controlled the weights of the average and squared gap in the cost function. Although BO tended to give inaccurate geometries by using too small or too large *α* values, all three optimization campaigns gave fairly accurate geometries by using appropriate *α* values ranging from 25 to 50 for both molecules.

In this study, we aimed to validate of our BO strategy; thus, the energy calculations on NISQ devices were substituted with DFT and TDDFT methods. We also located the GM and CI structures for both molecules with high accuracy, even when artificial random noise was added to the energies to simulate the geometry optimization using noisy quantum computer measurements. The application of our BO strategy for geometry optimization using the energies measured by the NISQ device will be reported in the future.

## Supporting Information

Additional supporting information can be found online in the Supporting Information section.

## Conflict of Interests

The authors declare no conflicts of interest.

5

## Supporting information

As a service to our authors and readers, this journal provides supporting information supplied by the authors. Such materials are peer reviewed and may be re‐organized for online delivery, but are not copy‐edited or typeset. Technical support issues arising from supporting information (other than missing files) should be addressed to the authors.

Supporting Information

## Data Availability

The data that support the findings of this study are available from the corresponding author upon reasonable request.

## References

[1] H. B. Schlegel, “Geometry optimization,” *Wiley Interdisciplinary Reviews: Computational Molecular Science 1* (**2011**): 790–809, 10.1002/wcms.34.

[2] A. Shajan, M. Manathunga, A. W. Götz, and K. M. Merz Jr., “Geometry Optimization: A Comparison of Different Open-Source Geometry Optimizers,” *Journal of Chemical Theory and Computation 19* (**2023**): 7533–7541, 10.1021/acs.jctc.3c00188. 37870541

[3] H. P. Hratchian, and H. B. Schlegel, *Theory and Applications of Computational Chemistry: The First 40 Years*, eds. C. E. Dykstra, G. Frenking, K. S. Kim, and G. Scuseria( Amsterdam: Elsevier, **2005**), 195.

[4] B. P. Lanyon, J. D. Whitfield, G. G. Gillett, M. E. Goggin, M. P. Almeida, I. Kassal, J. D. Biamonte, M. Mohseni, B. J. Powell, M. Barbieri, A. Asouru-Guzik, and A. G. White, “Towards quantum chemistry on a quantum computer,” *Nature Chemistry 2* (**2010**): 106–111, 10.1038/nchem.483. 21124400

[5] H. P. Paudel, M. Syamlal, S. E. Crawford, Y. -L. Lee, R. A. Shugayev, P. Lu, P. R. Ohodnicki, D. Mollot, and Y. Duan, “Quantum Computing and Simulations for Energy Applications: Review and Perspective,” *ACS Engineering Au 2* (**2022**): 151–196, 10.1021/acsengineeringau.1c00033.

[6] K. Head-Marsden, J. Flick, C. J. Ciccarino, and P. Narang, “Quantum Information and Algorithms for Correlated Quantum Matter,” *Chemical Reviews 121* (**2021**): 3061–3120, 10.1021/acs.chemrev.0c00620. 33326218

[7] Y. Shikano, H. C. Watanabe, K. M. Nakanishi, and Y. Ohnishi, “Post-Hartree–Fock method in quantum chemistry for quantum computer,” *The European Physical Journal Special Topics 230* (**2021**): 1037–1051, 10.1140/epjs/s11734-021-00087-z.

[8] B. Bauer, S. Bravyi, M. Motta, and G. K.-L. Chan, “Quantum Algorithms for Quantum Chemistry and Quantum Materials Science,” *Chemical Reviews 120* (**2020**): 12685–12717, 10.1021/acs.chemrev.9b00829. 33090772

[9] Y. Cao, J. Romero, J. P. Olson, M. Degroote, P. D. Johnson, M. Keiferová, I. D. Kivlichan, T. Menke, B. Peropadre, N. P. D. Sawaya, S. Sim, L. Veis, and A. Aspuru-Guzik, “Quantum Chemistry in the Age of Quantum Computing,” *Chemical Reviews 119* (**2019**): 10856–10915, 10.1021/acs.chemrev.8b00803. 31469277

[10] K. Bharti, A. Cervera-Lierta, T. H. Kyaw, T. Haug, S. Alperin-Lea, A. Anand, M. Degroote, H. Heimonen, J. S. Kottmann, T. Menke, W. -K. Mok, S. Sim, L. -C. Kwek, and A. Aspuru-Guzik, “Noisy intermediate-scale quantum algorithms,” *Reviews of Modern Physics 94* (**2022**): 1–69, 10.1103/RevModPhys.94.015004.

[11] A. Callison, and N. Chancellor, “Hybrid quantum-classical algorithms in the noisy intermediate-scale quantum era and beyond,” *Physical Review A 106* (**2022**): 1–17, 10.1103/PhysRevA.106.010101.

[12] B. Shahriari, K. Swersky, Z. Wang, R. P. Adams, N. D. Freitas, *Proc. IEEE* **2016**, *104*, 148–175.

[13] C. E. Rasmussen, and C. K. I. Williams, *Gaussian Processes for Machine Learning* (Cambridge: The MIT Press, **2006**), 1–248.

[14] H. J. Kushner, “A New Method of Locating the Maximum Point of an Arbitrary Multipeak Curve in the Presence of Noise,” *Journal of Basic Engineering 86* (**1964**): 97–106, 10.1115/1.3653121.

[15] P. Auer, *Journal of Machine Learning Research 3* (**2003**): 397–422.

[16] L. Fang, E. Makkonen, M. Todorović, P. Rinke, and X. Chen, “Efficient Amino Acid Conformer Search with Bayesian Optimization,” *Journal of Chemical Theory and Computation 17* (**2021**): 1955–1966, 10.1021/acs.jctc.0c00648. PMC8023666 33577313

[17] L. Chan, G. R. Hutchison, and G. M. Morris, “Bayesian optimization for conformer generation,” *Journal of Cheminformatics 11* (**2019**): 1–11, 10.1186/s13321--019--0354--7. PMC6528340 31115707

[18] L. Chan, G. R. Hutchison, and G. M. Morris, “BOKEI: Bayesian optimization using knowledge of correlated torsions and expected improvement for conformer generation,” *Physical Chemistry Chemical Physics 22* (**2020**): 5211–5219, 10.1039/C9CP06688H. 32091055

[19] S. Bae, D. Shin, H. Kim, J. W. Han, and J. M. Lee, “Accelerated Structural Optimization for the Supported Metal System Based on Hybrid Approach Combining Bayesian Optimization with Local Search,” *Journal of Chemical Theory and Computation* 20 (**2024**): 2284–2296, 10.1021/acs.jctc.3c01265. 38358319

[20] S. Maeda, T. Taketsugu, and K. Morokuma, “Exploring transition state structures for intramolecular pathways by the artificial force induced reaction method,” *Journal of Computational Chemistry 35* (**2014**): 166–173, 10.1002/jcc.23481. 24186858

[21] S. Maeda, Y. Harabuchi, M. Takagi, T. Taketsugu, and K. Morokuma, “Artificial Force Induced Reaction (AFIR) Method for Exploring Quantum Chemical Potential Energy Surfaces,” *Chemical Record 16* (**2016**): 2232–2248, 10.1002/tcr.201600043. 27258568

[22] C. Bannwarth, S. Ehlert, and S. Grimme, “GFN2-xTB – An Accurate and Broadly Parametrized Self-Consistent Tight-Binding Quantum Chemical Method with Multipole Electrostatics and Density-Dependent Dispersion Contributions,” *Journal of Chemical Theory and Computation 15* (**2019**): 1652–1671, 10.1021/acs.jctc.8b01176. 30741547

[23] S. Maeda, Y. Harabuchi, M. Takagi, K. Saita, K. Suzuki, T. Ichino, Y. Sumiya, K. Sugiyama, and Y. Ono, “Implementation and performance of the artificial force induced reaction method in the GRRM17 program,” *Journal of Computational Chemistry 39* (**2018**): 233–251, 10.1002/jcc.25106. PMC5765425 29135034

[24] S. Maeda, Y. Harabuchi, Y. Sumiya, M. Takagi, K. Suzuki, M. Hatanaka, Y. Osada, T. Taketsugu, K. Morokuma, K. Ohno, GRRM20, see http://iqce.jp/GRRM/index_e.shtml (accecced date 11 April, 2023). This notation is based on the online manual of this software (https://afir.sci.hokudai.ac.jp/manual/grrm17/grrm17_52.html).

[25] S. Maeda, K. Ohno, and K. Morokuma, “Systematic exploration of the mechanism of chemical reactions: the global reaction route mapping (GRRM) strategy using the ADDF and AFIR methods,” *Physical Chemistry Chemical Physics 15* (**2013**): 3683–3701, 10.1039/c3cp44063j. 23389653

[26] F. Neese, F. Wennmohs, U. Becker, and C. Riplinger, “The ORCA quantum chemistry program package,” *The Journal of Chemical Physics 152* (**2020**): 1–18, 10.1063/5.0004608. 32534543

[27] J. -D. Chai, and M. H. Gordon, “Long-range corrected hybrid density functionals with damped atom–atom dispersion corrections,” *Physical Chemistry Chemical Physics 10* (**2008**): 6615–6620, 10.1039/b810189b. 18989472

[28] T. H. Dunning Jr., *The Journal of Chemical Physics 90* (**1989**): 1007–1023.

[29] M. J. Frisch, G. W. Trucks, H. B. Schlegel, G. E. Scuseria, M. A. Robb, J. R. Cheeseman, G. Scalmani, V. Barone, G. A. Petersson, H. Nakatsuji, X. Li, M. Caricato, A. Marenich, J. Bloino, B. G. Janesko, R. Gomperts, B. Mennucci, H. P. Hratchian, J. V. Ortiz, A. F. Izmaylov, J. L. Sonnenberg, D. Williams-Young, F. Ding, F. Lipparini, F. Egidi, J. Goings, B. Peng, A. Petrone, T. Henderson, D. Ranasinghe, V. G. Zakrzewski, J. Gao, N. Rega, G. Zheng, W. Liang, M. Hada, M. Ehara, K. Toyota, R. Fukuda, J. Hasegawa, M. Ishida, T. Nakajima, Y. Honda, O. Kitao, H. Nakai, T. Vreven, K. Throssell, J. A. Montgomery, Jr., J. E. Peralta, F. Ogliaro, M. Bearpark, J. J. Heyd, E. Brothers, K. N. Kudin, V. N. Staroverov, T. Keith, R. Kobayashi, J. Normand, K. Raghavachari, A. Rendell, J. C. Burant, S. S. Iyengar, J. Tomasi, M. Cossi, J. M. Millam, M. Klene, C. Adamo, R. Cammi, J. W. Ochterski, R. L. Martin, K. Morokuma, O. Farkas, J. B. Foresman, and D. J. Fox, *Gaussian 09*, Revision E.01, Gaussian, Inc., Wallingford CT, USA, **2016**.

[30] S. Maeda, Y. Harabuchi, T. Taketsugu, and K. Morokuma, “Systematic Exploration of Minimum Energy Conical Intersection Structures near the Franck–Condon Region,” *Journal of Physical Chemistry A 118* (**2014**): 12050–12058, 10.1021/jp507698m. 25259835

[31] S. Maeda, K. Ohno, and K. Morokuma, “Automated Global Mapping of Minimal Energy Points on Seams of Crossing by the Anharmonic Downward Distortion Following Method: A Case Study of H_2_CO,” *Journal of Physical Chemistry A 113* (**2009**): 1704–1710, 10.1021/jp810898u. 19183041

[32] D. P. Kngma, J. L. Ba, **2014**, arXiv:1412.6980, 10.48550/arXiv.1412.6980.

[33] J. R. Gardner, G. Pleiss, D. Bindel, K. Q. Weinberger, A. G. Wilson, **2018**, arXiv:1809.11165, 10.48550/arXiv.1809.11165.

[34] M. Balandat, B. Karrer, D. R. Jiang, S. Daulton, B. Letham, A. G. Wilson, E. Bakshy, **2019**, arXiv:1910.06403, 10.48550/arXiv.1910.06403.

[35] R. H. Byrd, P. Lu, J. Nocedal, and C. Zhu, “A Limited Memory Algorithm for Bound Constrained Optimization,” *SIAM Journal on Scientific Computation 16* (**1995**): 1190–1208, 10.1137/0916069.

[36] C. Zhu, R. H. Byrd, P. Lu, and J. Nocedal, “Algorithm 778: L-BFGS-B,” *ACM Transactions on Mathematical Software 23* (**1997**): 550–560, 10.1145/279232.279236.

[37] Y. Shao, M. H. Gordon, and A. I. Krylov, “The spin–flip approach within time-dependent density functional theory: Theory and applications to diradicals,” *The Journal of Chemical Physics 118* (**2003**): 4807–4818, 10.1063/1.1545679.

[38] N. Minezawa, and M. S. Gordon, “Optimizing Conical Intersections by Spin−Flip Density Functional Theory: Application to Ethylene,” *Journal of Physical Chemistry A 113* (**2009**): 12749–12753, 10.1021/jp908032x. 19905013

[39] Y. Harabuchi, M. Hatanaka, and S. Maeda, *npj Comput Mater* **2019**, *9*, 1–8, 10.1038/s41524-023-00965-1.

[40] S. Gocho, H. Nakamura, S. Kanno, Q. Gao, T. Kobayashi, T. Inagaki, and M. Hatanaka, *npj Computational Materials 13* (**2023**): 1–9.

