# Supplementary material for: Exploration of the Global Minimum and Conical Intersection with Bayesian Optimization
Source: Mol Inform. 2025 Jan 30;44(2):e202400041. doi: 10.1002/minf.202400041 (PMC11781018; doi:10.1002/minf.202400041)
Supplement: Supplementary file 1 — Supporting Information [file MINF-44-e202400041-s001.pdf]

Molecular Informatics

Supporting Information

Exploration of the Global Minimum and Conical Intersection with Bayesian Optimization

Riho Somaki[1] | Taichi Inagaki[1] | Miho Hatanaka[1,2]

Molecular Informatics

Supporting Information

Exploration of the Global Minimum and Conical Intersection with Bayesian Optimization

Riho Somaki[1] | Taichi Inagaki[1] | Miho Hatanaka[1,2]

Supporting Information for

Exploration of the Global Minimum and Conical Intersection with Bayesian Optimization

Riho Somaki,[†] Taichi Inagaki,[†] and Miho Hatanaka[†, ‡,*]

[†]*Graduate School of Science and Technology, Keio University. 3-14-1 Hiyoshi, Kohoku-ku, Yokohama, Kanagawa, 223-8522, Japan.*

[‡]*Institute for Molecular Science.38 NishigoNaka, Myodaiji, Okazaki, Aichi, 444-8585, Japan.*

**E-mail: hatanaka@chem.keio.ac.jp*

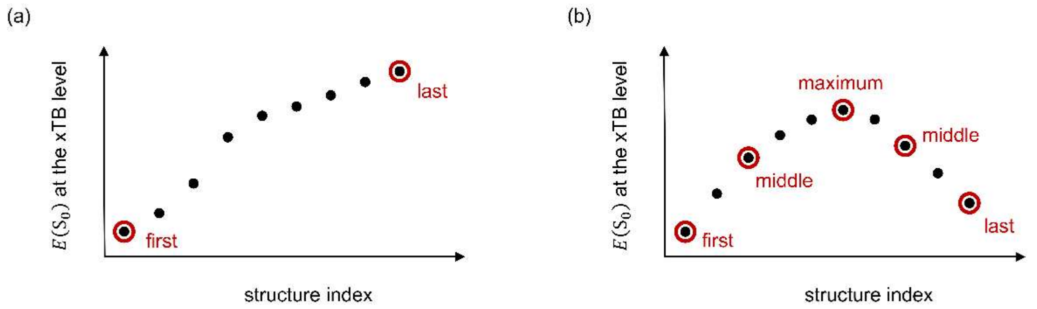

Figure S1. The method to extract geometries for initial training dataset from each path obtained by the SC-AFIR*n* method in step 0. Each path consists of geometries and their energies shown in black dots. When $E(S_0)$ increases or decreases along the path, we extract two structures in red (a). If the path has an up-convex shape in $E(S_0)$, we extract five structures in red (Note that "middle" represents the structure that has the closest $E(S_0)$ to the average of $E(S_0)$ at the first/last and the maximum points) (b). The shapes of the paths (a) and (b) can be recognized as follows: if $E(S_0)$ of the first or last structure on the path coincides with $E(S_0)$ maximum of the structure on the path, the shape of the path is determined to be (a), otherwise (b).

Table S1. List of 12 AFs examined for the CI search of formaldehyde. [a]

Entry	AFs [b]
1	$\mathrm{PI}(x; y = E(\mathrm{S}_0)) \times \mathrm{PI}(x; y = E(\mathrm{S}_1) - E(\mathrm{S}_0))$
2	$\mathrm{PI}(x; y = E(\mathrm{S}_0)) \times \mathrm{PI}(x; y = E(\mathrm{S}_1)) \times \mathrm{PI}(x; y = E(\mathrm{S}_1) - E(\mathrm{S}_0))$
3	$\mathrm{PI}(x; y = E(\mathrm{S}_0)) \times \mathrm{PI}(x; y = \lvert E(\mathrm{S}_1) - E(\mathrm{S}_0)\rvert)$
4	$\mathrm{PI}(x; y = E(\mathrm{S}_0)) \times \mathrm{PI}(x; y = E(\mathrm{S}_1)) \times \mathrm{PI}(x; y = \lvert E(\mathrm{S}_1) - E(\mathrm{S}_0)\rvert)$
5	$\mathrm{PI}(x; y = E(\mathrm{S}_1)) \times \mathrm{PI}(x; y = E(\mathrm{S}_1) - E(\mathrm{S}_0))$
6	$\mathrm{PI}(x; y = E(\mathrm{S}_1)) \times \mathrm{PI}(x; y = \lvert E(\mathrm{S}_1) - E(\mathrm{S}_0)\rvert)$
7	$\mathrm{PI}(x; y = E(\mathrm{S}_0)) + \mathrm{PI}(x; y = E(\mathrm{S}_1) - E(\mathrm{S}_0))$
8	$\mathrm{PI}(x; y = E(\mathrm{S}_0)) + \mathrm{PI}(x; y = E(\mathrm{S}_1)) + \mathrm{PI}(x; y = E(\mathrm{S}_1) - E(\mathrm{S}_0))$
9	$\mathrm{PI}(x; y = E(\mathrm{S}_0)) + \mathrm{PI}(x; y = \lvert E(\mathrm{S}_1) - E(\mathrm{S}_0)\rvert)$
10	$\mathrm{PI}(x; y = E(\mathrm{S}_0)) + \mathrm{PI}(x; y = E(\mathrm{S}_1)) + \mathrm{PI}(x; y = \lvert E(\mathrm{S}_1) - E(\mathrm{S}_0)\rvert)$
11	$\mathrm{PI}(x; y = E(\mathrm{S}_1)) + \mathrm{PI}(x; y = E(\mathrm{S}_1) - E(\mathrm{S}_0))$
12	$\mathrm{PI}(x; y = E(\mathrm{S}_1)) + \mathrm{PI}(x; y = \lvert E(\mathrm{S}_1) - E(\mathrm{S}_0)\rvert)$

[a] All of them did not meet the termination criterion before 1000 iterations.

[b] $\mathrm{PI}(x; y = f)$ represents the probability of improvement, in which the objective variable y was defined as f.

Algorithm 1 select 200 structures from random 2000 structures

1. Generate 2000 structures χ_k $(k = 1, \cdots, 2000)$ randomly using the quasi-Monte Calro method.
2. Calculate the AF value of each structure using the GPR model:

$$\mathrm{AF}(\chi_k)\ (k = 1, \cdots, 2000)$$

3. Standardize $\mathrm{AF}(\chi_k)$ $(k = 1, \cdots, 2000)$:

$$\mathrm{AF_{st}}(\chi_k)\ (k = 1, \cdots, 2000)$$

4. Calculate the exponential of the standardized AF value:

$$\exp(\mathrm{AF_{st}}(\chi_k))\ (k = 1, \cdots, 2000)$$

5. Select 200 structures based on $\exp(\mathrm{AF_{st}}(\chi_k))$ to reproduce its distribution:

$$\hat{\chi}_j\ (j = 1, \cdots, 200)$$

Figure S2. The algorithm for selecting 200 structures from random 2000 structures in step 2. In this procedure, AF values were calculated without checking whether all the atomic distances are smaller than 0.5 Å or not.

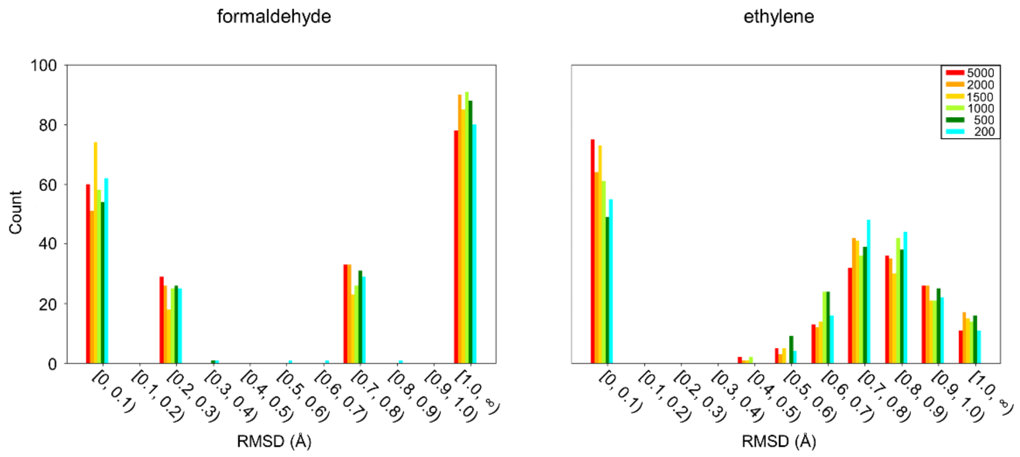

Figure S3. The histogram of the selected 200 structures from randomly generated *m* structures (*m* = 200, 500, 1000, 1500, 2000, 5000) at the first iteration of BO with the algorithm in Figure S2. The horizontal and vertical axes represent the range of RMSD of the generated structures from the reference (true) GM structure and their numbers, respectively. The data distribution of the selected structures was almost independent of *m*, which indicated that *m* = 2000 in our procedure was large enough.

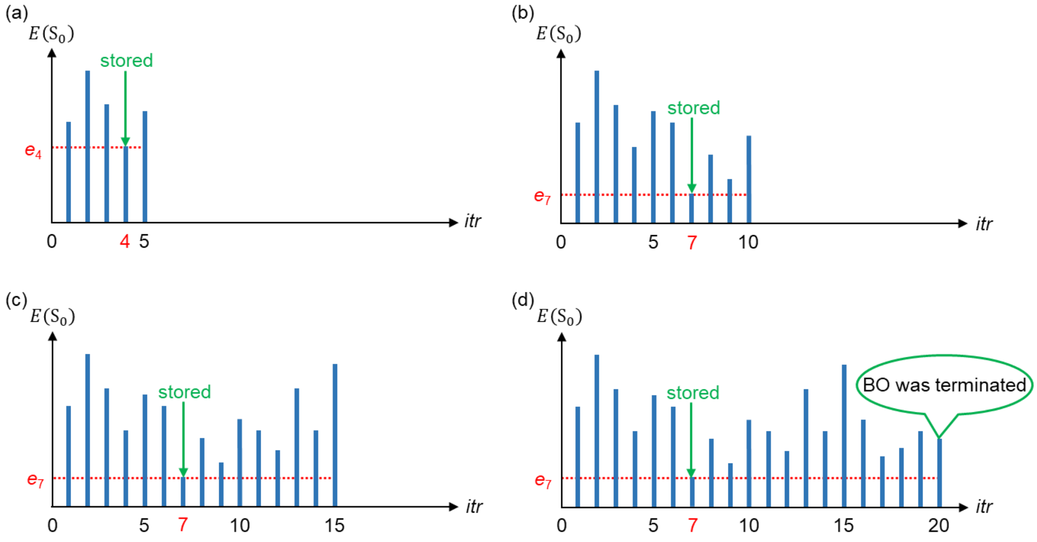

Figure S4. The schematic illustration of the termination criterion for the GM search. The horizontal and vertical axes represent the iteration number (*itr*) of BO and the ground state energy calculated at the candidate geometry with the DFT method ($E(S_0)$). (a)~(d) shows how the stored structure changes as *itr* increases. After the 5 iterations of BO (*itr* = 5), the data of the lowest energy among the five was e_4 at *itr* = 4, thus its geometry and energy e_4 were stored (a). After the 10 iterations of BO, the lowest energy among the ten was e_7 at *itr* = 7, thus, the stored data was updated from that at *itr* = 4 to that at *itr* = 7 (b). After the 15 iterations, the lowest energy among the fifteen was still e_7, thus, the stored data was not updated (c). After the 20 iterations, the lowest energy among the twenty was still e_7, and the stored data was not updated again (d). BO was terminated at *itr* = 20, because the stored data was not updated in three consecutive checks at *itr* = 10, 15, 20.

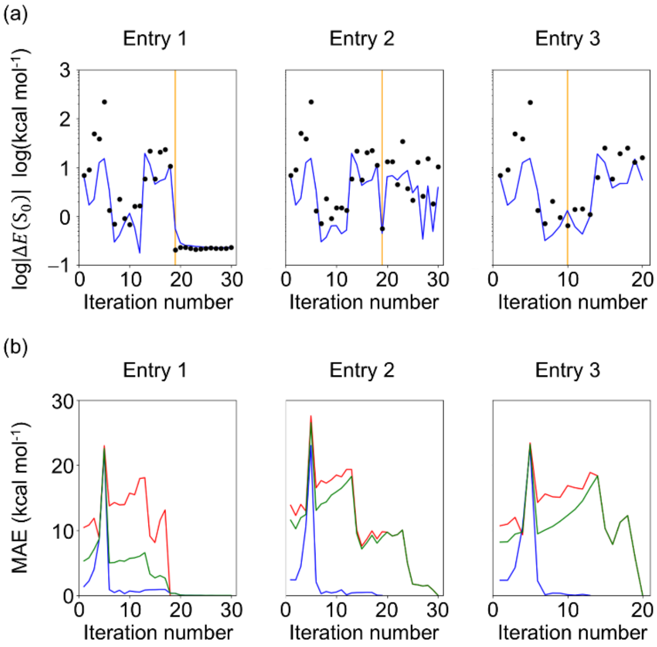

Figure S5. The deviation of the candidate geometry from the reference GM geometry of formaldehyde at each iteration for entries 1, 2, and 3 in Table 1. The absolute energy differences $|\Delta E(S_0)|$ in log scale calculated with the DFT and predicted from the GPR model are represented by black dots and a blue line, respectively (a). The evolution of the mean absolute errors (MAEs) on the $E(S_0)$ prediction of all future candidates, future candidates close to the reference GM (RMSD < 0.03 Å), and future candidates far from the reference GM (RMSD ≥ 0.03 Å) are shown in green, blue, and red, respectively (b). The energy differences and RMSD are shown in Figure 5.

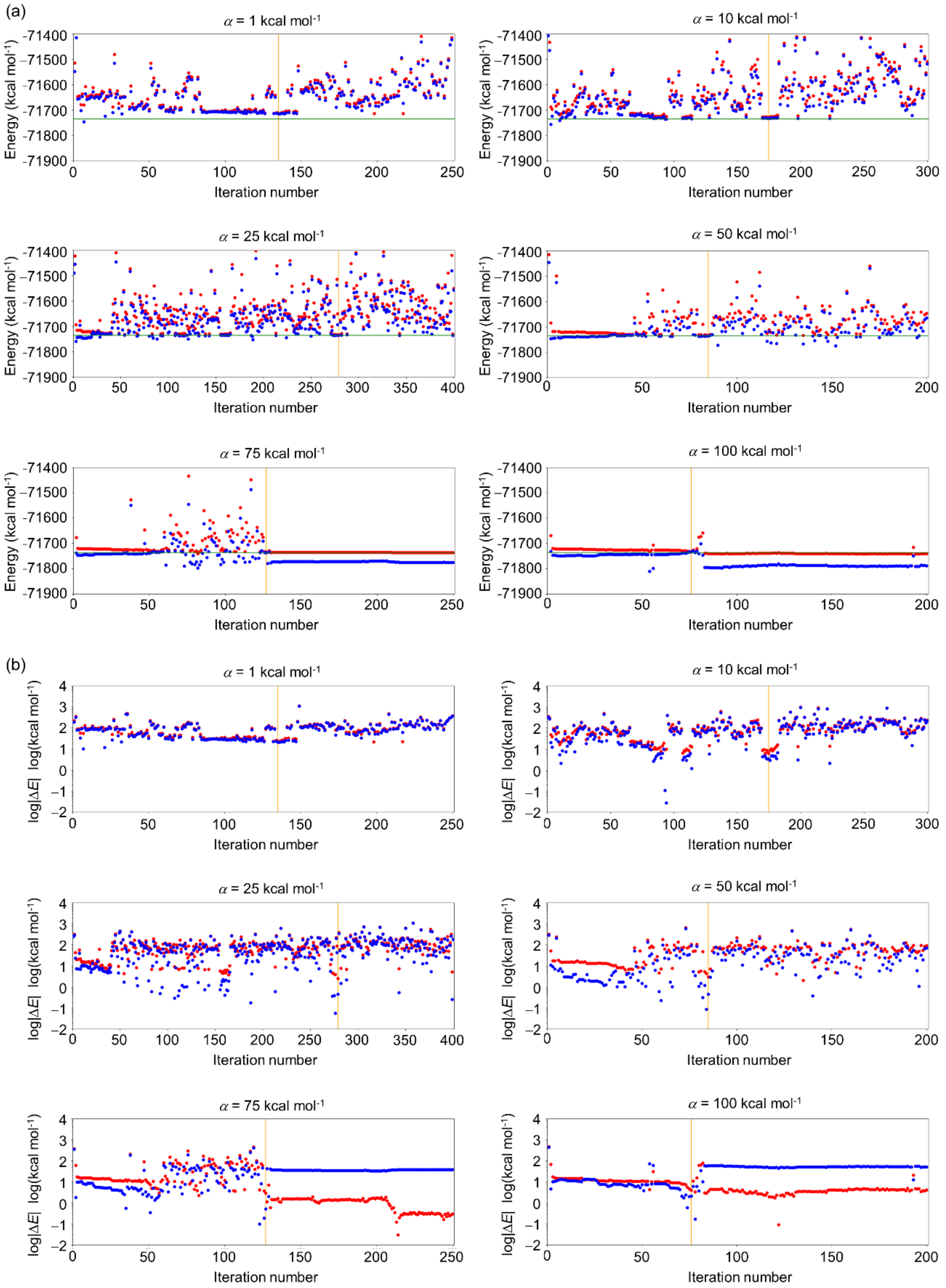

Figure S6. The $E(S_0)$ and $E(S_1)$ (in kcal mol^{-1}) of the candidate geometry generated at each iteration in the CI search of formaldehyde. The BO campaigns of entries 1, 4, 7, 10, 13, and 16 in Table 2 are shown as representative of BO with $\alpha = 1$, 10, 25, 50, 75, and 100 kcal mol^{-1}, respectively. The energy in the rage from −71900 to −71400 kcal mol^{-1} is shown for better visibility.The $E(S_0)$ at the reference CI geometry is shown in green line (a). The log-scale plots for the absolute energy difference from the $E(S_0)$ of the reference CI are displayed (b).

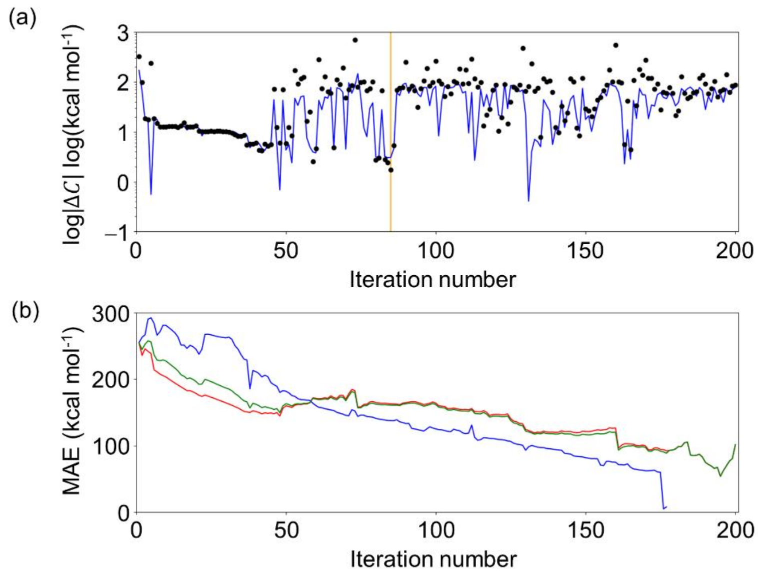

Figure S7. The deviation of the cost function C (in kcal mol^{-1}) from the reference CI geometry for formaldehyde at each iteration for entry 10 in Table 2. The absolute cost function difference $|\Delta C|$ in log scale calculated with the DFT/TDDFT and predicted from the GPR model are represented by black dots and a blue line, respectively (a). The evolution of the MAEs on the C prediction of all future candidates, future candidates close to the reference CI (RMSD < 0.2 Å), and future candidates far from the reference CI (RMSD $\geq$ 0.2 Å) are shown in green, blue, and red, respectively (b). The ΔC, RMSD, $E(S_0)$, and $E(S_1)$ are shown in Figure 7.

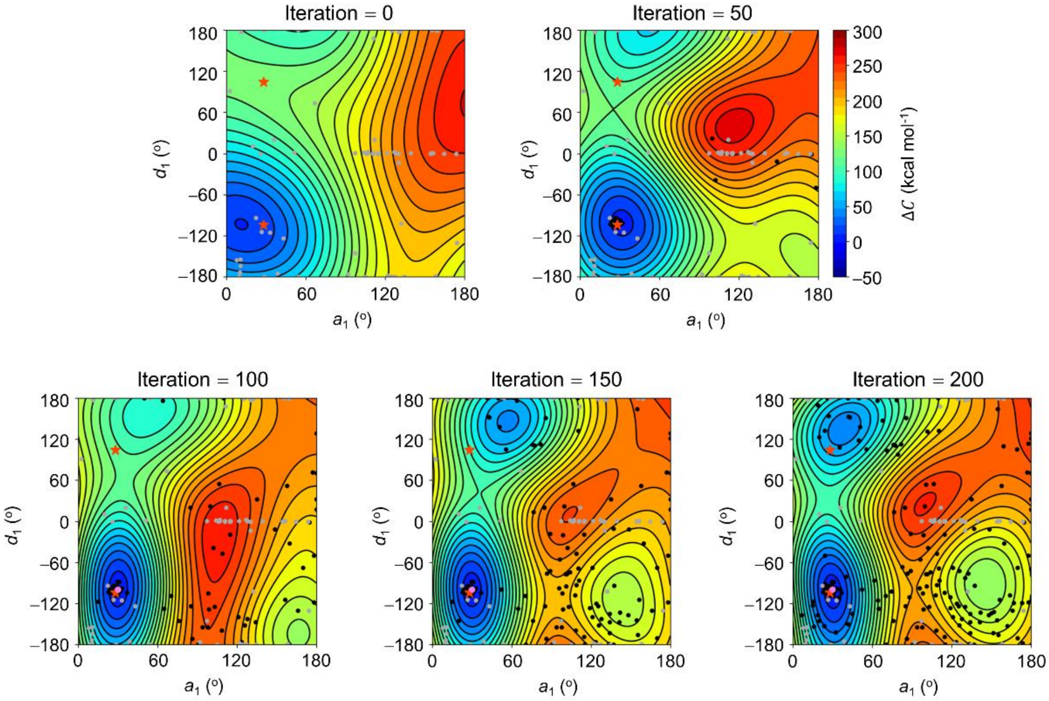

Figure S8. The evolution of the cost function surface (CFS) for the CI search of formaldehyde as iteration number of BO increased. The CFSs are measured from the C value at the reference CI geometry. The CFSs before and after BO are the same as Figure 8c and 8d, respectively. The initial, candidate, and optimized geometries are in gray, black, and light pink dots, respectively. The reference geometries are in orange stars.

Table S2. Results on the GM search of formaldehyde using UCB.

Entry	β	Optimization campaign [a]	Iteration number [b]	Energy difference $\Delta E(S_0)$ (kcal mol^{-1}) [c]	RMSD (Å) [c]
1	0.01	1	30 (19)	0.131	0.0121
2		2	– [d]	–	–
3		3	– [d]	–	–
4	0.1	1	70 (59)	0.234	0.0104
5		2	45 (34)	0.254	0.0100
6		3	55 (45)	0.254	0.0093
7	0.5	1	20 (9)	6.038	0.0652
8		2	20 (9)	6.044	0.0652
9		3	20 (9)	6.050	0.0653
10	1	1	20 (9)	24.789	0.1620
11		2	20 (9)	24.890	0.1622
12		3	20 (9)	25.058	0.1628

[a] We performed three optimization campaigns because BO procedure involves the randomness in step 2. The optimization campaign IDs (1, 2, and 3) were named in order of decreasing the $\Delta E(S_0)$.
[b] Number of iterations until termination of BO. The numbers in parentheses are the iteration numbers that yielded the lowest-energy geometry.
[c] Energy difference $\Delta E(S_0)$ and RMSD were calculated from the reference GM structure.
[d] The calculation was manually terminated since only geometries with atomic collisions were generated repeatedly in step 2.

Table S3. Results on the most stable CI search of formaldehyde using the UCB.

Entry	β	α (kcal mol^{-1})	Optimization campaign [a]	Iteration number [b]	Energy difference $\Delta E(S_0)$ (kcal mol^{-1}) [c]	Energy difference $\Delta E(S_1)$ (kcal mol^{-1}) [c]	Energy gap $E(S_1)-E(S_0)$ (kcal mol^{-1})	RMSD (Å) [c]
1	0.01	1	1	200 (100)	17.568	18.952	1.576	0.0819
2			2	200 (63)	75.399	75.429	0.221	0.5794
3			3	300 (179)	78.867	80.841	2.165	0.5724
4		10	1	400 (258)	6.035	6.620	0.776	0.0644
5			2	150 (16)	12.118	14.670	2.742	0.0867
6			3	150 (16)	12.124	14.715	2.782	0.0867
7		25	1	300 (158)	2.214	2.243	0.220	0.0989
8			2	300 (168)	3.057	3.126	0.260	0.1113
9			3	200 (82)	14.788	17.292	2.695	0.1098
10		50	1	1000 (992) [d]	0.425	2.068	1.834	0.0667
11			2	1000 (943) [d]	1.385	2.753	1.559	0.0756
12			3	900 (779)	8.427	8.967	0.731	0.1468
13		75	1	1000 (950) [d]	0.249	1.206	1.148	0.0363
14			2	1000 (892)	1.956	2.845	1.080	0.0780
15			3	1000 (951) [d]	39.429	40.054	0.816	0.1683
16		100	1	1000 (985) [d]	0.702	1.770	1.259	0.0247
17			2	900 (797)	1.182	1.353	0.362	0.0614
18			3	1000 (×) [e]	–	–	–	–
19	0.1	1	1	250 (138)	46.671	47.129	0.649	0.2209
20			2	400 (253)	48.381	48.580	0.389	0.3171
21			3	300 (158)	74.172	76.719	2.738	0.5400

22		10	1	550 (404)	0.629	1.117	0.679	0.0547
23			2	450 (305)	3.309	3.596	0.479	0.0604
24			3	550 (449)	3.986	3.848	0.053	0.1011
25		25	1	500 (356)	1.104	1.151	0.239	0.0236
26			2	450 (323)	2.109	2.299	0.380	0.0955
27			3	200 (98)	2.227	2.281	0.245	0.0909
28		50	1	1000 (901) [d]	0.534	2.557	2.213	0.0291
29			2	1000 (951) [d]	2.198	4.469	2.462	0.0268
30			3	1000 (960) [d]	23.103	25.763	2.850	0.5503
31		75	1	1000 (879)	0.472	2.089	1.809	0.0496
32			2	300 (186)	0.637	0.671	0.225	0.0445
33			3	1000 (902) [d]	1.224	1.171	0.137	0.0654
34		100	1	1000 (856)	33.261	34.614	1.544	0.3456
35			2	1000 (×) [e]	–	–	–	–
36			3	1000 (×) [e]	–	–	–	–
37	0.5	1	1	300 (195)	77.163	78.596	1.623	0.4478
38			2	150 (45)	91.244	92.770	1.717	0.4005
39			3	200 (63)	168.083	169.569	1.677	0.5313
40		10	1	800 (690)	10.806	11.301	0.686	0.0876
41			2	400 (281)	17.283	17.997	0.905	1.2098
42			3	800 (674)	27.320	27.795	0.666	0.2691
43		25	1	400 (279)	1.352	1.459	0.298	0.0737
44			2	650 (530)	1.616	1.777	0.352	0.0196
45			3	300 (187)	9.859	11.531	1.863	0.1149
46		50	1	700 (583)	0.342	0.471	0.320	0.0293
47			2	700 (589)	0.783	0.836	0.245	0.0635
48			3	950 (840)	20.090	21.959	2.060	0.2191
49		75	1	300 (164)	0.503	0.703	0.391	0.0211
50			2	350 (240)	0.956	1.014	0.249	0.0367
51			3	950 (823)	65.929	67.580	1.842	0.5779
52		100	1	1000 (965) [d]	1.365	3.961	2.787	0.0438
53			2	1000 (935) [d]	1.906	4.040	2.325	0.0918
54			3	1000 (×) [e]	–	–	–	–
55	1	1	1	200 (57)	59.155	60.945	1.981	0.3985
56			2	200 (92)	69.343	71.843	2.692	0.5212
57			3	150 (42)	131.057	133.666	2.801	0.3080
58		10	1	350 (233)	48.050	49.476	1.618	0.2881
59			2	250 (113)	93.756	95.739	2.174	0.3234
60			3	250 (112)	141.160	143.813	2.844	0.5343
61		25	1	450 (326)	14.723	16.176	1.645	0.6084
62			2	550 (406)	22.312	24.850	2.729	0.2161
63			3	200 (85)	111.342	112.641	1.491	0.5495
64		50	1	350 (210)	17.283	19.519	2.427	0.1775
65			2	200 (61)	104.684	106.316	1.823	0.4542
66			3	550 (432)	123.456	125.335	2.069	0.6499
67		75	1	150 (26)	40.122	42.015	2.084	0.2497
68			2	150 (26)	40.373	41.747	1.565	0.2497
69			3	450 (311)	119.841	120.302	0.651	0.5976

70	100	1	900 (797)	23.805	26.520	2.906	0.1274
71		2	1000 (966) [d]	35.646	36.288	0.834	0.2664
72		3	1000 (×) [e]	–	–	–	–

[a] We performed three optimization campaigns because BO procedure involves the randomness in step 2. The optimization campaign IDs (1, 2, and 3) were named in order of decreasing the $\Delta E(S_0)$.
[b] Number of iterations until the termination of BO. The numbers in parentheses are the iteration numbers that yielded the lowest-C geometry.
[c] Energy difference and RMSD were calculated from the reference CI structure.
[d] Though the termination criterion was not met, the structure stored at the end of BO is shown.
[e] The termination criterion was not met and none of the structures was stored during BO.

Table S4. Results on the GM search of ethylene.

Entry [a]	Iteration number [b]	Energy difference $\Delta E(S_0)$ (kcal mol^{-1}) [c]	RMSD (Å) [c]
1	35 (23)	0.368	0.0140
2	25 (14)	1.357	0.0345
3	15 (5)	1.660	0.0301

[a] We performed three optimization campaigns because the BO procedure involves randomness in step 2.
[b] Number of iterations until termination of BO. The numbers in parentheses are the iteration numbers that yielded the lowest-energy geometry.
[c] Energy difference $\Delta E(S_0)$ and RMSD were calculated from the reference GM structure.

Table S5. Results on the most stable CI search of ethylene.

Entry	α (kcal mol^{-1})	Optimization campaign [a]	Iteration number [b]	Energy difference $\Delta E(S_0)$ (kcal mol^{-1}) [c]	Energy difference $\Delta E(S_1)$ (kcal mol^{-1}) [c]	Energy gap $E(S_1)$–$E(S_0)$ (kcal mol^{-1})	RMSD (Å) [c]
1	1	1	200 (54)	7.231	8.411	1.400	0.6503
2		2	150 (48)	7.646	8.941	1.514	0.6619
3		3	150 (41)	8.310	8.893	0.802	0.6692
4	10	1	300 (199)	3.340	4.251	1.130	0.0807
5		2	250 (143)	6.257	6.596	0.558	0.0967
6		3	150 (9)	7.383	8.455	1.291	0.6559
7	25	1	250 (135)	1.399	1.316	0.136	0.1332
8		2	450 (330)	1.477	2.189	0.931	0.0470
9		3	450 (330)	2.081	3.288	1.426	0.1139
10	50	1	300 (200)	0.709	1.933	1.442	0.0421
11		2	350 (235)	1.437	1.904	0.686	0.1201
12		3	500 (353)	2.321	2.666	0.564	0.0603
13	75	1	300 (161)	1.816	2.236	0.639	0.0652
14		2	150 (24)	6.602	9.165	2.782	0.6361
15		3	150 (12)	6.799	9.197	2.616	0.6580
16	100	1	250 (142)	1.877	1.799	0.141	0.0813
17		2	650 (502)	2.949	3.318	0.589	0.0733
18		3	150 (3)	6.550	8.753	2.422	0.6509

[a] We performed three optimization campaigns because BO procedure involves the randomness in step 2. The optimization campaign IDs (1, 2, and 3) were named in order of decreasing the $\Delta E(S_0)$.
[b] Number of iterations until the termination of BO. The numbers in parentheses are the iteration numbers that yielded the lowest-C geometry.
[c] Energy difference and RMSD were calculated from the reference CI structure.

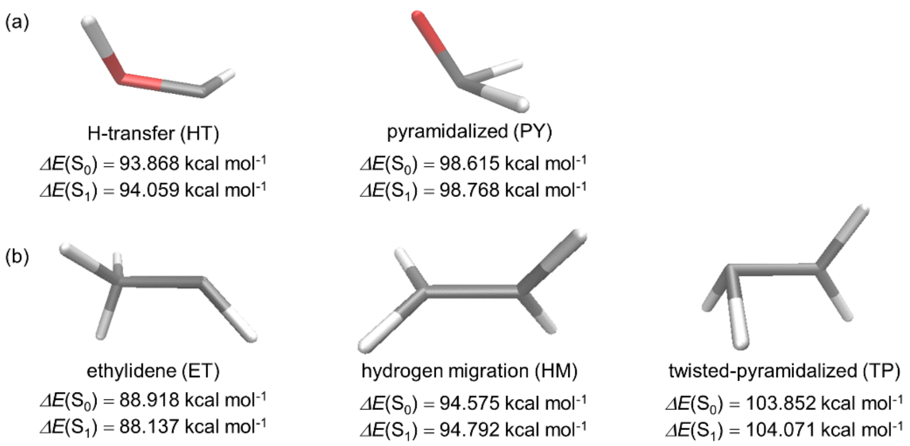

Figure S9. The CI geometries and their energy differences (in kcal mol^{-1}) from the GM of formaldehyde (a) and ethylene (b) optimized at the DFT/TDDFT (ωB97XD/cc-pVDZ) level combined with the energy shift approximation. The energy shift parameter was 0.20 kcal mol^{-1}.

Table S6. Results on the GM search with random energy noise of formaldehyde.

Entry [a]	Iteration number [b]	Energy difference $\Delta E(S_0)$ (kcal mol^{-1}) [c]	RMSD (Å) [c]
1	20 (8)	−0.049	0.0317
2	15 (4)	0.870	0.0364
3	15 (3)	0.915	0.0343

[a] We performed three optimization campaigns because the BO procedure involves randomness in step 2.
[b] Number of iterations until termination of BO. The numbers in parentheses are the iteration numbers that yielded the lowest-energy geometry.
[c] Energy difference $\Delta E(S_0)$ and RMSD were calculated from the reference GM structure.

Table S7. Results on the GM search with random energy noise of ethylene.

Entry [a]	Iteration number [b]	Energy difference $\Delta E(S_0)$ (kcal mol^{-1}) [c]	RMSD (Å) [c]
1	25 (13)	−1.408	0.0091
2	30 (16)	0.445	0.0306
3	25 (15)	1.441	0.0528

[a] We performed three optimization campaigns because the BO procedure involves randomness in step 2.
[b] Number of iterations until termination of BO. The numbers in parentheses are the iteration numbers that yielded the lowest-energy geometry.
[c] Energy difference $\Delta E(S_0)$ and RMSD were calculated from the reference GM structure.

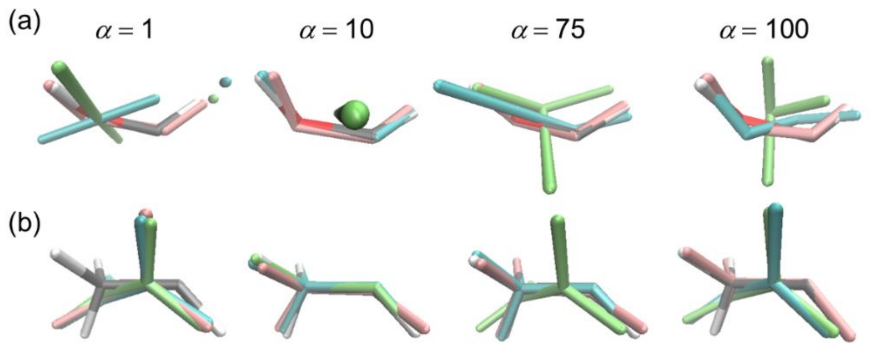

Figure S10. Comparison between the reference geometry (in the same atom color code as in Figure 2) and the geometries obtained *via* the first, second, and third BO campaigns (in pink, blue, and green, respectively) in the most stable CI search with $\alpha = 1$, 10, 75, and 100 kcal mol^{-1}, for foraldehyde (a) and ethylene (b). The results with $\alpha = 25$ and 50 kcal mol^{-1} are in Figure 10.

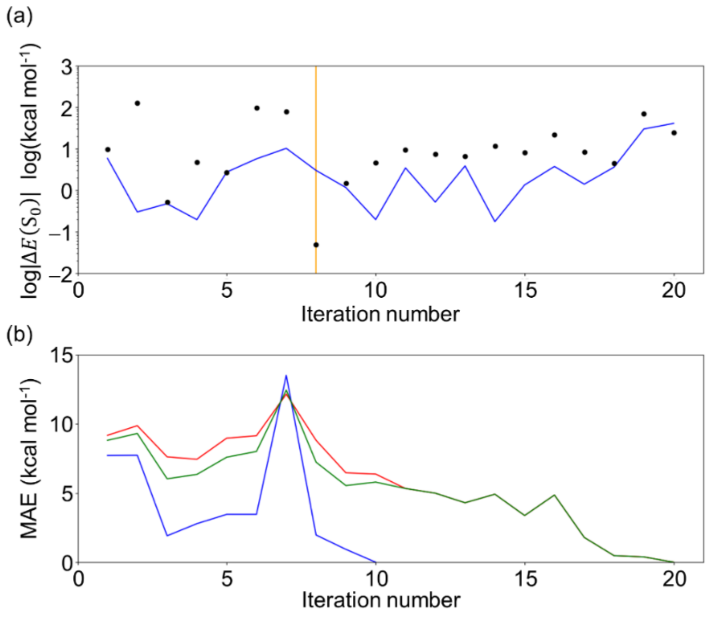

Figure S11. The deviation of the candidate geometry from the reference GM of formaldehyde at each iteration for entry 1 in Table S6. The absolute energy errors $|\Delta E(S_0)|$ in log scale calculated with the DFT and predicted from the GPR model are represented by black dots and a blue line, respectively (a). The evolution of the MAEs on the $E(S_0)$ prediction of all future candidates, future candidates close to the reference GM (RMSD < 0.04 Å), and future candidates far from the reference GM (RMSD $\geq$ 0.04 Å) are shown in green, blue, and red, respectively (b). The energy errors and RMSD are shown in Figure 11.

Table S8. Results on the most stable CI search with random energy noise of formaldehyde.

Entry	α (kcal mol^{-1})	Optimization campaign [a]	Iteration number [b]	Energy difference $\Delta E(S_0)$ (kcal mol^{-1}) [c]	Energy difference $\Delta E(S_1)$ (kcal mol^{-1}) [c]	Energy gap $E(S_1)–E(S_0)$ (kcal mol^{-1})	RMSD (Å) [c]
1	1	1	300 (198)	37.440	37.424	0.175	0.1493
2		2	450 (311)	43.234	43.922	0.879	1.2024
3		3	350 (221)	76.999	78.049	1.241	0.5577
4	10	1	350 (214)	5.512	6.984	1.663	0.1174
5		2	200 (94)	8.125	10.520	2.587	0.0500
6		3	400 (281)	17.622	18.508	1.077	1.2131
7	25	1	200 (99)	1.387	4.097	2.902	0.0714
8		2	200 (82)	1.521	1.520	0.190	0.0389
9		3	150 (34)	3.650	5.761	2.302	0.0717
10	50	1	500 (388)	0.461	1.776	1.506	0.0194
11		2	300 (175)	0.625	2.439	2.005	0.0443
12		3	150 (24)	5.539	7.716	2.369	0.0757
13	75	1	250 (108)	1.480	1.897	0.608	0.0484
14		2	550 (406)	39.603	41.961	2.549	0.3104
15		3	400 (285)	152.415	153.623	1.399	0.5610
16	100	1	200 (57)	3.202	5.440	2.430	0.0667
17		2	250 (144)	13.757	13.863	0.297	0.0541
18		3	500 (362)	33.557	35.148	1.782	0.6921

[a] We performed three optimization campaigns because BO procedure involves the randomness in step 2. The optimization campaign IDs (1, 2, and 3) were named in order of decreasing the $\Delta E(S_0)$.
[b] Number of iterations until the termination of BO. The numbers in parentheses are the iteration numbers that yielded the lowest-C geometry.
[c] Energy difference and RMSD were calculated from the reference CI structure.

Table S9. Results on the most stable CI search with random energy noise of ethylene.

Entry	α (kcal mol[-1])	Optimization campaign [a]	Iteration number [b]	Energy difference $\Delta E(S_0)$ (kcal mol[-1]) [c]	Energy difference $\Delta E(S_1)$ (kcal mol[-1]) [c]	Energy gap $E(S_1)-E(S_0)$ (kcal mol[-1])	RMSD (Å) [c]
1	1	1	250(123)	10.589	12.683	2.312	0.6547
2		2	150(25)	11.742	13.124	1.601	0.6335
3		3	150(23)	21.195	23.863	2.887	0.6551
4	10	1	400(290)	4.615	5.411	1.014	0.1981
5		2	300(151)	4.802	6.077	1.494	0.0961
6		3	300(188)	5.403	6.034	0.850	0.0852
7	25	1	400(268)	0.198	1.168	1.190	0.0519
8		2	300(158)	1.811	3.280	1.688	0.0592
9		3	400(280)	2.613	3.249	0.855	0.1110
10	50	1	450(321)	0.410	2.978	2.787	0.0535
11		2	200(57)	1.614	3.155	1.760	0.0489
12		3	300(192)	1.676	2.814	1.357	0.1680
13	75	1	700(589)	0.913	2.101	1.407	0.2099
14		2	250(143)	3.366	3.723	0.576	0.0522
15		3	150(36)	6.833	7.612	0.998	0.6607
16	100	1	500(395)	1.731	1.890	0.378	0.0656
17		2	150(17)	6.790	9.079	2.508	0.6484
18		3	200(51)	7.904	8.806	1.121	0.6424

[a] We performed three optimization campaigns because BO procedure involves the randomness in step 2. The optimization campaign IDs (1, 2, and 3) were named in order of decreasing the $\Delta E(S_0)$.
[b] Number of iterations until the termination of BO. The numbers in parentheses are the iteration numbers that yielded the lowest-C geometry.
[c] Energy difference and RMSD were calculated from the reference CI structure.

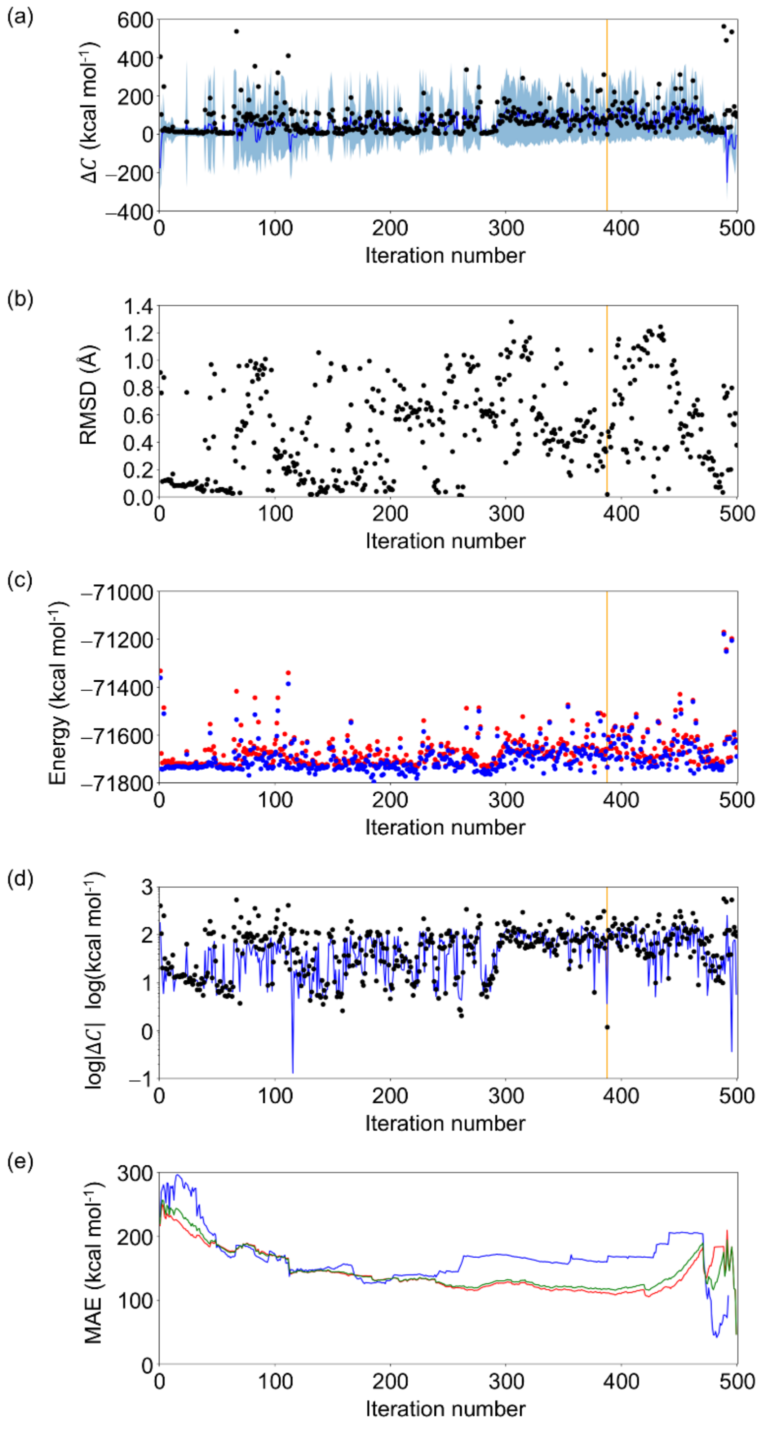

Figure S12. The deviation of the cost function ΔC (in kcal mol^{-1}) with random noises from the reference CI geometry for formaldehyde. The candidate geometries for the CI search were from entry 10 in Table S8 (α = 50 kcal mol^{-1}). The orange lines represent the iteration number that yielded the optimized geometry. The ΔC values calculated by the DFT/TDDFT level with noises and the GPR model are in black and blue, respectively. The 68% confidence interval of the GPR model is in light blue. (a) The deviation of the candidate geometry from the reference CI geometry was estimated by the RMSD (in Å) (b) and the S_0 and S_1 energies (in blue and red, respectively) at the DFT/TDDFT level with noises (c). The absolute cost function difference $|\Delta C|$ in log scale calculated with the DFT/TDDFT and predicted from the GPR model are represented by black dots and a blue line, respectively (d). The evolution of the MAEs on the C prediction of all future candidates, future candidates close to the reference CI (RMSD < 0.2 Å), and future candidates far from the reference CI (RMSD $\geq$ 0.2 Å) are shown in green, blue, and red, respectively (e).
